# Abstinence-Induced Nicotine Seeking Relays on a Persistent Hypoglutamatergic State within the Amygdalo-Striatal Neurocircuitry

**DOI:** 10.1523/ENEURO.0468-22.2023

**Published:** 2023-02-20

**Authors:** Ana Domi, Esi Domi, Oona Lagstrom, Francesco Gobbo, Elisabet Jerlhag, Louise Adermark

**Affiliations:** 1 Institute of Neuroscience and Physiology, Department of Pharmacology, The Sahlgrenska Academy, University of Gothenburg, Gothenburg 413 90, Sweden; 2 Addiction Biology Unit, Department of Psychiatry and Neurochemistry, Institute of Neuroscience and Physiology, The Sahlgrenska Academy, University of Gothenburg, Gothenburg 413 45, Sweden; 3 School of Pharmacy, Pharmacology Unit, Center for Neuroscience, University of Camerino, Camerino 62032, Italy; 4 Centre for Discovery Brain Sciences, University of Edinburgh, Edinburgh EH8 9JZ, United Kingdom

**Keywords:** amygdala, electrophysiology, glutamate, nicotine relapse, striatum

## Abstract

Nicotine robustly sustains smoking behavior by acting as a primary reinforcer and by enhancing the incentive salience of the nicotine-associated stimuli. The motivational effects produced by environmental cues associated with nicotine delivery can progressively manifest during abstinence resulting in reinstatement of nicotine seeking. However, how the activity in reward neuronal circuits is transformed during abstinence-induced nicotine seeking is not yet fully understood. In here we used a contingent nicotine and saline control self-administration model to disentangle the contribution of cue-elicited seeking responding for nicotine after drug abstinence in male Wistar rats. Using *ex vivo* electrophysiological recordings and a network analysis approach, we defined temporal and brain-region specific amygdalo-striatal glutamatergic alterations that occur during nicotine abstinence. The results from this study provide critical evidence indicating a persistent hypoglutamatergic state within the amygdalo-striatal neurocircuitry over protracted nicotine abstinence. During abstinence-induced nicotine seeking, electrophysiological recordings showed progressive neuroadaptations in dorsal and ventral striatum already at 14-d abstinence while neuroadaptations in subregions of the amygdala emerged only after 28-d abstinence. The observed neuroadaptations pointed to a brain network involving the amygdala and the dorsolateral striatum (DLS) to be implied in cue-induced reinstatement of nicotine seeking. Together these data suggest long-lasting neuroadaptations that might reflect neuroplastic changes responsible to abstinence-induced nicotine craving. Neurophysiological transformations were detected within a time window that allows therapeutic intervention advancing clinical development of preventive strategies in nicotine addiction.

## Significance Statement

Most people attempting to quit smoking, experience multiple periods of remission and relapse within the first month of abstinence. Our results provide evidence of a persistent hypoglutamatergic state over four weeks of nicotine abstinence when assessing parallel changes in evoked glutamate transmission in multiple amygdalo-striatal subregions at once. The observed neuroadaptations pointed to a brain network involving the amygdala and the dorsolateral striatum (DLS) that correlated with cue-induced reinstatement of nicotine seeking. The importance of these findings resides in an improved picture when capturing a single frame of the amygdalo-striatal circuitry in abstinence-induced nicotine craving. The neurophysiological transformations were detected within a time window that allows therapeutic intervention advancing clinical development of preventive strategies in nicotine addiction.

## Introduction

Cigarette smoking and smokeless tobacco are the main responsible causes for the global tobacco epidemic that worldwide only in 2019 caused nearly 8 million deaths (Reitsma et al., 2021; World Health Organization 2021). Nicotine is the principal psychoactive component of cigarette smoke. Unlike other drugs such as psychostimulants or opioids, nicotine addiction is strongly driven by reinforcing sensory stimuli that through associative learning processes increase smoking behavior (Bevins and Palmatier, 2004; Baumann and Sayette, 2006; LaRowe et al., 2007). In both humans and animals, contextual salient sensory cues can outmatch the direct effects of nicotine as demonstrated by the persistence of responding for nicotine, increased intensity and time of smoking in absence of the drug (LeSage et al., 2004; Raiff and Dallery, 2008; Rose et al., 2010; Perkins et al., 2017).

The motivational effects produced by contextual visual cues associated with nicotine delivery can progressively manifest during prolonged abstinence, a phenomenon that refers to reinstatement of nicotine seeking and incubation of nicotine craving (Abdolahi et al., 2010; Funk et al., 2016; Markou et al., 2018). In smokers, responses to nicotine-related cues increase over time consistently with relapse vulnerability (Bedi et al., 2011). The craving phenomenon after drug cessation has been largely studied in animals identifying pharmacological treatment targets for relapse prevention with more robust results obtained after withdrawal from cocaine and heroin (for review, see Pickens et al., 2011; Venniro et al., 2016).

While there is very little evidence on the neurobiological adaptations that occur during prolonged abstinence of cue-induced nicotine seeking, the amygdala appears to be an important substrate for incubation of nicotine craving and relapse vulnerability (Goudriaan et al., 2010; Janes et al., 2010; Funk et al., 2016; Xue et al., 2017). Accordingly, nicotine-dependent subjects who fail to quit smoking, show greater cue-induced reactivity within the amygdala and dorsal striatum compared with those who remain abstinent, pointing to a critical role of these neuronal substrates in nicotine relapse vulnerability (Janes et al., 2010). Intriguingly, neurophysiological adaptations in the central and basolateral amygdalar nuclei CeA and BLA, respectively, elicited by psychostimulants have been shown to drive parallel dopamine-dependent transformations within striatal subregions through changes in glutamatergic neurotransmission (Murray et al., 2015). Amygdalo-striatal neuroadaptations have also been reported following protracted withdrawal from experimenter-controlled daily nicotine injections (Licheri et al., 2020). Progressive adaptations in amygdalo-striatal neurocircuits may thus reflect the increasing risk of nicotine relapse.

In the present study, we used a contingent nicotine and saline control self-administration model to define the contribution of cue-elicited seeking responding for nicotine after drug abstinence. Using *ex vivo* electrophysiological recordings and a network analysis approach, we defined temporal and brain-region specific alterations in glutamatergic neurotransmission in amygdalo-striatal circuits during abstinence from nicotine. Recordings were conducted within the first weeks of abstinence, that are described as the most critical for smokers that want to quit (Hughes, 2007).

## Materials and Methods

### Animals

Experiments were performed using male Wistar rats from Charles River weighing 200–220 g at arrival. Rats were paired-housed in a temperature and humidity-controlled environment under a reversed 12/12 h light/dark cycle (lights off at 8 A.M.) with food and water *ad libitum*. Rats were habituated to the facility and handled before experiments. Experiments were conducted during the dark phase of the cycle and all efforts were made to minimize rats’ suffering and distress. Procedures were conducted in accordance with the National Committee for Animal Research in Sweden and approved by the Local Ethics Committee for Animal Care and Use at Gothenburg University.

### Drugs

The (−)-Nicotine hydrogen tartrate salt (Sigma-Aldrich) was dissolved in sterile physiological saline (0.9% NaCl) and administered intravenously at a concentration of 30.0 μg/kg/0.1 ml infusion. The pH of the solution was adjusted to 7.4 with NaOH 5 m. The GABAA receptor antagonist bicuculline-methiodide (bicuculline) was diluted in Milli-Q to 20 mm and further diluted in artificial CSF (aCSF; 20 μm), while the AMPA receptor antagonist CNQX was dissolved in aCSF (10 μm) shortly before use. All drugs were purchased from Sigma-Aldrich.

### Catheter implantation

Animals were anesthetized with isoflurane anesthesia (Forene, Baxter: 5% induction and 2% maintenance, O_2_: 2 l/min). Analgesia during and after surgery was maintained with Metacam 1 mg/kg given by subcutaneous injections (5 mg/ml, Boehringer Ingelheim). A single catheter made from microrenathane tubing (ID = 0.020″, OD = 0.037″; Braintree Scientific) was implanted in the right jugular vein as previously described (Shen et al., 2017; Domi et al., 2021). Immediately after surgery and for the subsequent 3 d, rats were treated with 10 mg/kg of subcutaneous enrofloxacin (50 mg/ml, Baytril) and allowed to one week recovery before starting the self-administration training. Catheters were flushed daily throughout the experiment with 0.1–0.2 ml of sterile saline mixed with heparin (20 U/ml; Italfarmaco S.p.A) to maintain the catheter patency that was confirmed by intravenous injection of 150 μl/rat of Propofol-Lipuro (20 mg/ml, B. Braun Medical AB) at the end of the experimental procedures.

### Self-administration apparatus

Nicotine self-administration was performed in rat operant conditioning chambers (ENV-008CT Med Associates) enclosed in sound-attenuating, ventilated environmental cubicles. Each chamber was equipped with two 4-cm-wide retractable levers located in the front panel with two stimulus light placed above each lever, a house light at the top of the opposite panel and a tone generator. Nicotine and saline solutions were delivered by Tygon tubing connected to a swivel and then to the catheter before the beginning of each session. A syringe pump (3.33 RPM, Med Associates) was activated by the response on the active lever according to the programmed schedule, while responses on the inactive lever were recorded but had no scheduled consequence. Activation of the pump resulted in the delivery of 0.1 ml of fluid. The operant chambers were controlled, and data were collected with MED-PC IV windows-compatible software.

### Experimental procedures

#### Nicotine self-administration

##### Acquisition and maintenance under fixed ratio

**Figure 1.**
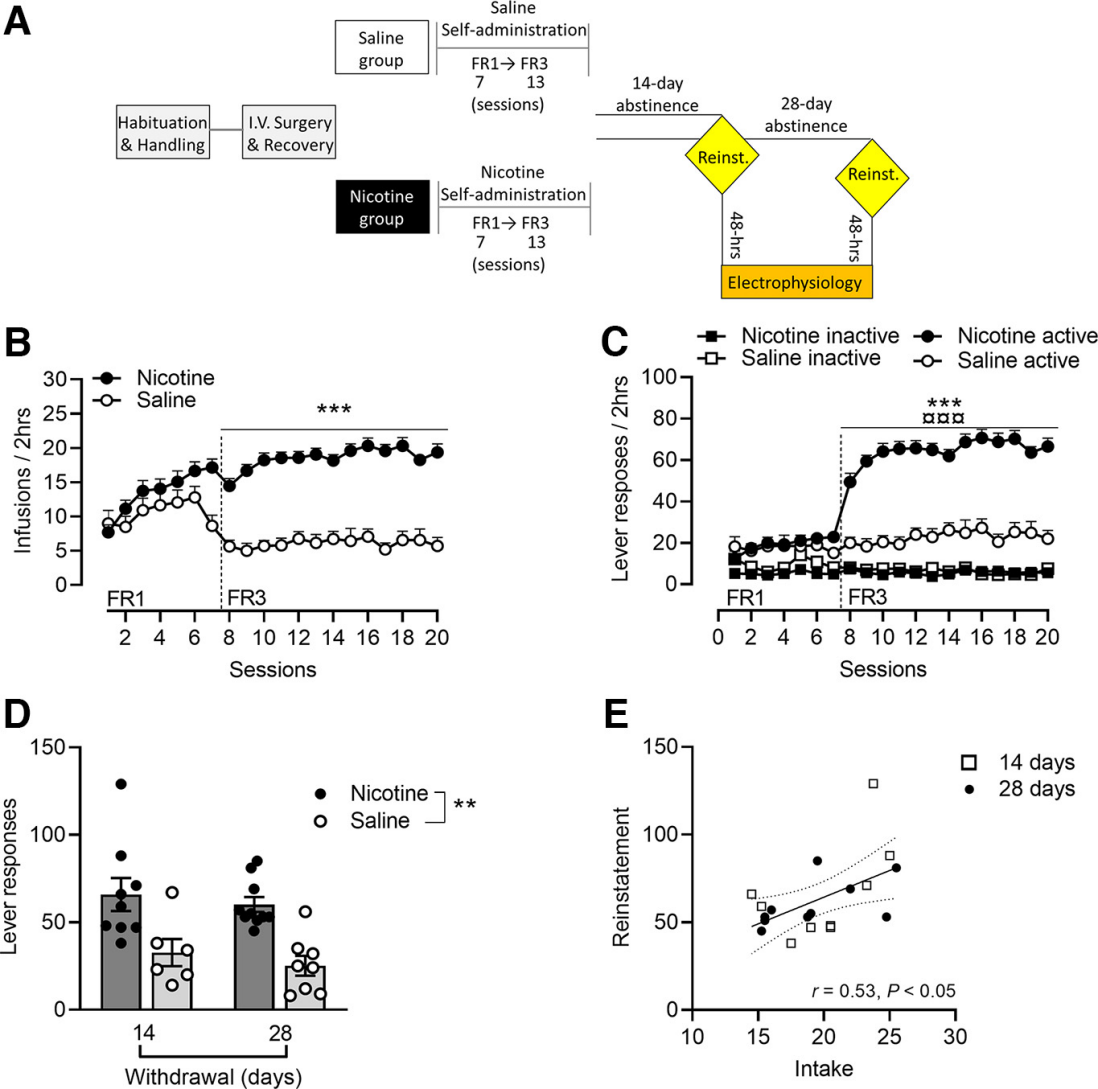
Nicotine self-administration and relapse to nicotine seeking. ***A***, Timeline of the experimental procedures that include behavioral and neurophysiological assessments. ***B***, The Acquisition pattern of self-administration showed a significantly higher number of self-infusions and active lever responses (***C***) in the nicotine rats (*n* = 19) compared with the saline control group (*n* = 14). Extended Data Figure 1-1 shows baseline responding during training for nicotine and saline rats in the 14-d abstinence and 28-d abstinence group. ***D***, Nicotine rats showed similar levels of responding in cue-induced nicotine seeking in the 14-d abstinence and 28-d abstinence group, exhibiting significant reinstatement levels as compared with rats trained with saline. Extended Data Figure 1-2 shows time course of active lever responding of cue-induced reinstatement of nicotine seeking (1-min bins of time) in the 14- and 28-d abstinence group. ***E***, Positive correlation of the magnitude of cue-induced reinstatement with the number of responses during nicotine training. Values are presented as mean (±SEM), ***p < 0.001 significant as compared with nicotine and saline rats in the number of reinforcers and active lever presses (days 8–20), ¤¤¤*p* < 0.001 (active lever vs inactive lever) in nicotine rats. **p < 0.01 significant as compared with nicotine and saline rats in reinstatement responding.

A week after surgery rats (≈65–70 d old) were randomly assigned to self-administer either nicotine (30.0 μg/kg/0.1 ml infusion) or saline (0.9% NaCl) for 2 h/d (5 d/week) under fixed ratio-1 (FR1) schedule of reinforcement. Seven days of FR1 schedule were used for the rats to acquire operant learning. After 7 d, 89.5% of rats reached an acquisition criterion of ≥10 nicotine infusions per session. The response requirement for each infusion was then incremented to FR3 to ensure stable nicotine self-administration rates for the remainder of the training (Fig. 1*A*). The delivery of nicotine or saline solution was accompanied by the illumination of the cue-light for 5-s above the active lever which corresponds to the time in which the total volume of 0.1 ml of solution was delivered by the infusion pump. A 20-s time out period (TO) where responses at the active lever were not reinforced followed each infusion. The entire duration of the 120-min session was signaled by the intermittent cue-tone (1 s ON/1 s OFF; 7 kHz, 70 dB) as previously described (Cippitelli et al., 2015; Wu et al., 2017).

10.1523/ENEURO.0468-22.2023.f1-1 Extended Data Figure 1-1 Baseline responding during training (average last 5 d) for nicotine and saline rats in the 14-d abstinence and 28-d abstinence group. Student’s *t* test showed no significant difference in the number of active lever presses in the14-d abstinence and 28-d abstinence nicotine group (*t*_(17)_ = 0.30, *p* = 0.77) and saline group (*t*_(12)_ = 0.79, *p* = 0.44). Download Figure 1-1, TIF file.

**Figure 2.**
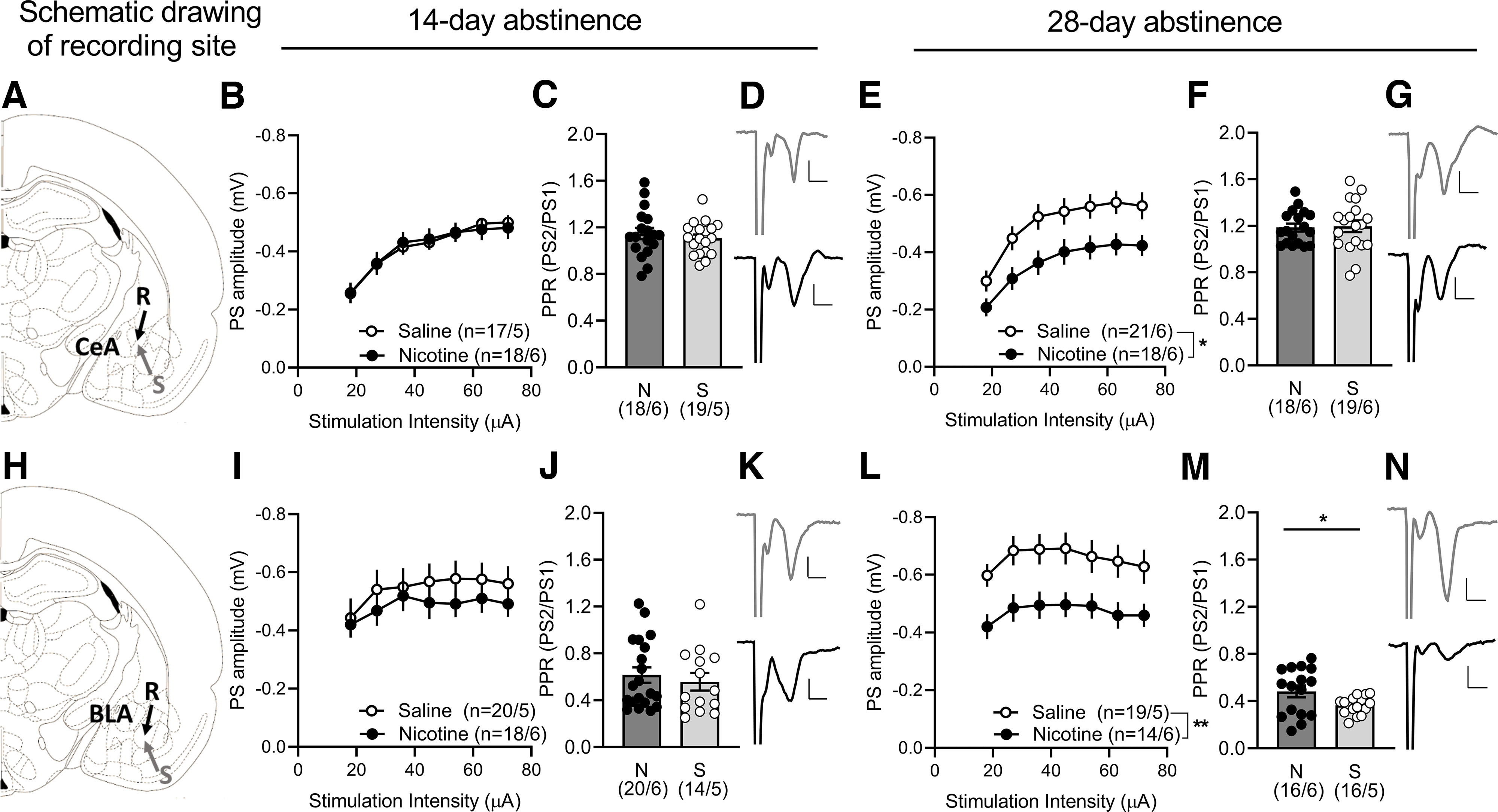
Neurophysiological transformations in the amygdala during abstinence-induced nicotine seeking. ***A***, ***H***, Schematic drawing showing the locations for field potential recordings conducted in the CeA and BLA. ***B***, ***I***, At 14-d abstinence we did not find differences in the input/output (I/O) function and in the paired pulse ratio (PPR; ***C***, ***J***) when comparing nicotine and saline rats in neither the CeA nor the BLA. ***E***, ***L***, Input/output function was significantly depressed at 28-d abstinence in both CeA and BLA in the nicotine group. ***F***, ***M***, The decrease in input/output was concomitant with a change in paired pulse ratio (PPR) in the BLA but not the CeA. ***D***, ***K***, Example traces (average 5 consecutive traces) showing evoked potentials in the CeA and BLA at 14-d abstinence and (***G***, ***N***) 28-d abstinence in brain slices from nicotine (black) and saline (gray) rats. Extended Data Figure 2-1 shows example traces of evoked PSs during input/output (I/O) function and paired-pulse stimulation (PPR) in BLA and CEA. Calibration: 0.2 mV, 2 ms. Values are presented as mean (±SEM), **p < 0.01, *p < 0.05 significant as compared with nicotine and saline rats (*n* = x/y; x: number of recordings/y: number of rats recorded).

10.1523/ENEURO.0468-22.2023.f1-2 Extended Data Figure 1-2 Time course of active lever responding of cue-induced reinstatement of nicotine seeking (1-min bins of time) in the 14- and 28-d abstinence group. When comparing lever responding in the first 10 min (1–10´) versus the last 10 min (111–120´) overall ANOVA revealed a significant effect of time (*F*_(1,17)_ = 27.66, *p* < 0.001), but not abstinence group (*F*_(1,17)_ = 0.85, *p* = 0.36) and time × abstinence group interaction (*F*_(1,17)_ = 3.31, *p* = 0.087). Download Figure 1-2, TIF file.

##### Abstinence and cue-induced reinstatement

After reaching a stable baseline each nicotine and saline subject was randomly assigned to the two subgroups designated as the 14- or 28-d abstinence groups, referring to the length of their withdrawal period before the cue-induced reinstatement and electrophysiological measures. During abstinence, rats were housed in the animal facility and handled three times per week. After this period animals were returned to the self-administration boxes to be tested for cue-induced reinstatement of nicotine seeking. Rats that were trained to self-administer saline underwent cue-induced reinstatement at the same test session conditions as the nicotine group. During the 2-h reinstatement session, responding at the active lever led to the activation of the cue light previously paired with the intravenous infusion but nicotine or saline were no longer delivered. The total number of responses on the lever previously associated to nicotine (active responses) was considered as a measure of reinstatement. Inactive lever presses were also recorded as a measure of nonspecific responding. Two rats were excluded from the study for catheter failure, and one rat was removed for nonsuccess to acquire nicotine self-administration.

### Electrophysiology

#### Brain slice preparation

Electrophysiological recordings were conducted starting from 48 h after reinstatement of cue-induced nicotine seeking. Coronal brain slices were prepared from nicotine and saline self-administering rats as previously described (Lagström et al., 2019; Licheri et al., 2020). In brief, rats were deeply anesthetized with isoflurane and euthanized. Brains were rapidly removed and transferred into a constantly oxygenated (95% O_2_, 5% CO_2_) modified artificial CSF (aCSF) containing (in mm): 220 sucrose, 2 KCl, 0.2 CaCl_2_, 6 MgCl_2_, 26 NaHCO_3_, 1.3 NaH_2_PO_4_, and 10 D-glucose. Coronal brain slices (250 μm) were obtained using a Leica VT 1200S Vibratome (Leica Microsystems) and submerged in a continuously oxygenated standard aCSF containing (in mm): 124 NaCl, 4.5 KCl, 2 CaCl_2_, 1 MgCl_2_, 26 NaHCO_3_, 1.2 NaH_2_PO_4_, and 10 D-glucose. After an incubation for 30 min in 33°C, slices were allowed to rest for additionally 30 min in room temperature before electrophysiological recordings were performed. Slices were maintained at room temperature for the rest of the day.

#### Field potential recordings

**Figure 3.**
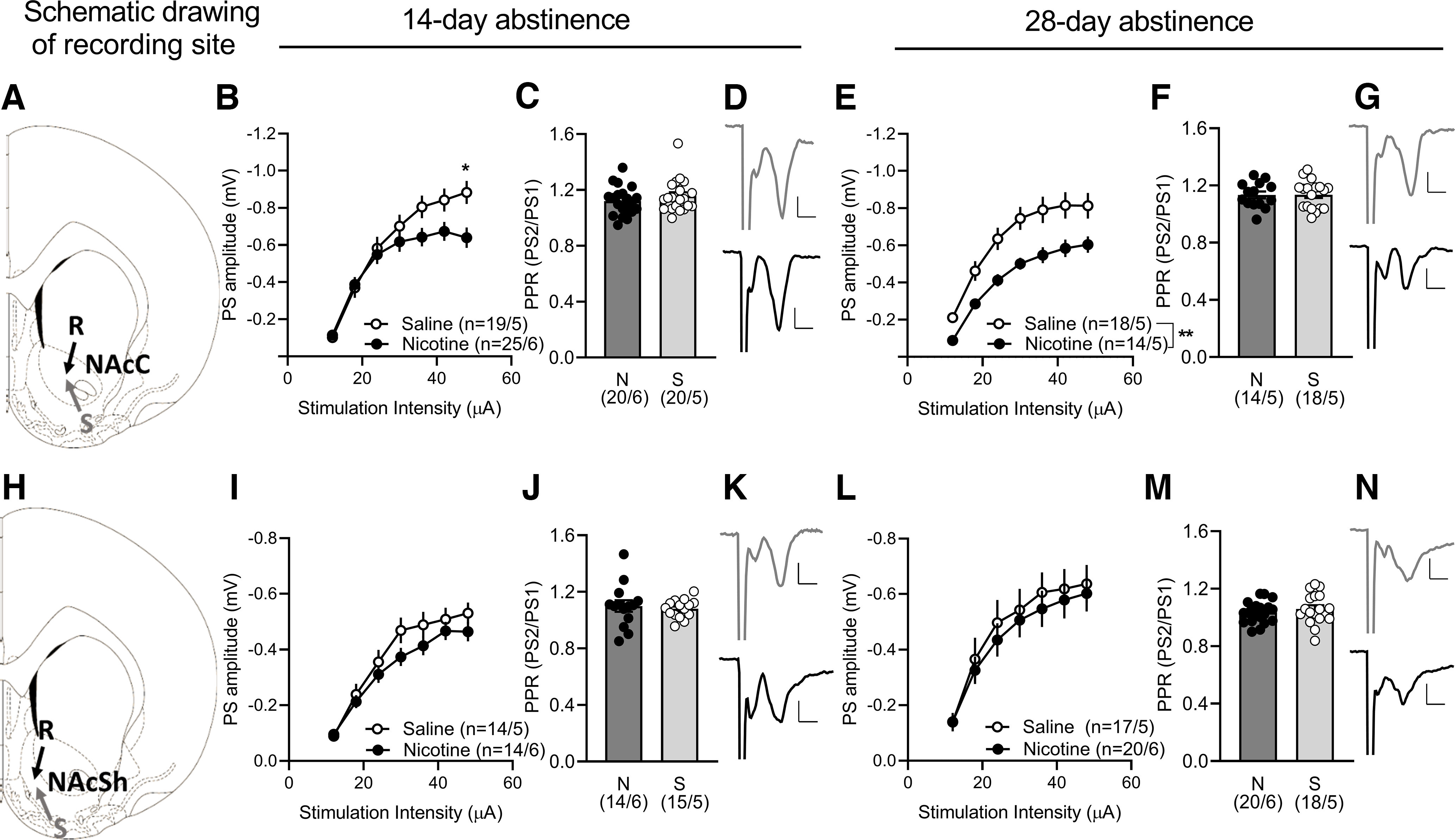
Neurophysiological transformations in the accumbens during abstinence-induced nicotine seeking. ***A***, ***H***, Schematic drawing showing the locations for field potential recordings conducted in the NAcC and NAcSh. ***B***, ***E***, In the NAcC a decrease in the input/output function in nicotine rats was apparent from 14-d abstinence and progressed at the second abstinence point while ***C***, ***F***, PPR was not significantly modulated by nicotine. ***I***, ***L***, In the NAcSh stimulus response curves produced no significant differences in the amplitude of evoked field potentials. ***J***, ***M***, There was also no effect influenced by treatment on PPR. ***D***, ***K***, Example traces (average five consecutive traces) showing evoked potentials in the NAcC and NAcSh at 14-d abstinence and (***G***, ***N***) 28-d abstinence in brain slices from nicotine (black) and saline (gray) rats. Extended Data Figure 3-1 shows example traces of evoked PSs during input/output (I/O) function and paired-pulse stimulation (PPR) in NAcC and NAcSh. Calibration: 0.2 mV, 2 ms. Values are presented as mean (±SEM), **p < 0.01, *p < 0.05 significant as compared with nicotine and saline rats (*n* = x/y; x: number of recordings/y: number of rats recorded).

**Figure 4.**
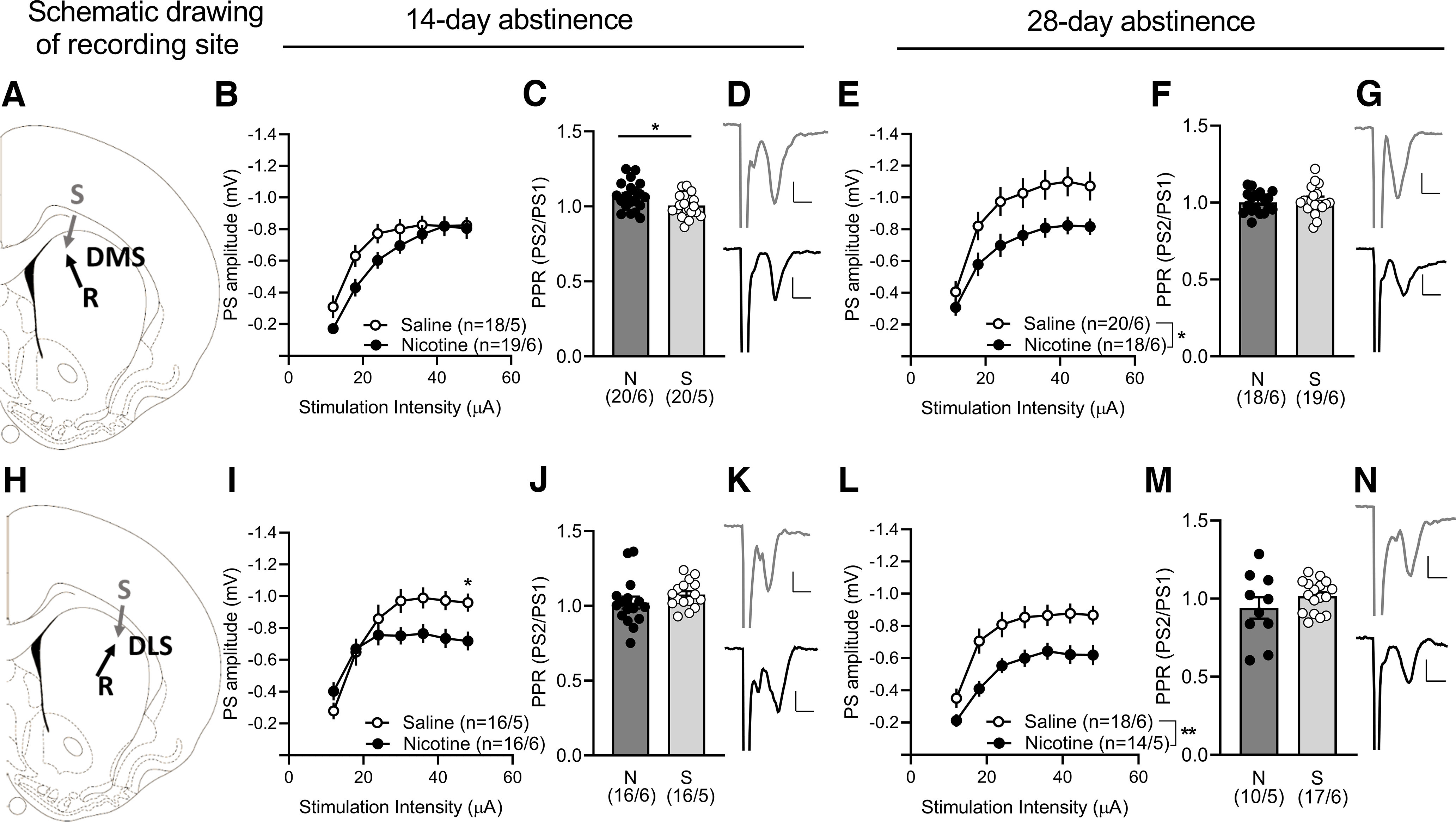
Neurophysiological transformations in the dorsal striatum during abstinence-induced nicotine seeking. ***A***, ***H***, Schematic drawing showing the locations for field potential recordings conducted in the DMS and DLS. ***B***, ***E***, In the DMS a decrease in the input/output function in nicotine rats was apparent from 14-d abstinence and progressed at the second withdrawal point. ***C***, ***F***, PPR was significantly modulated by nicotine in the14-d abstinence only. ***I***, ***L***, In the DLS the input/output function was significantly depressed at higher stimulation strengths after 14-d abstinence, and then more robustly at 28-d abstinence while PPR (***J***, ***M***) was not significantly modulated by nicotine. ***D***, ***K***, Example traces (average five consecutive traces) showing evoked potentials in the DMS and DLS at 14-d abstinence and (***G***, ***N***) 28-d abstinence in brain slices from nicotine (black) and saline (gray) rats. Extended Data Figure 4-1 shows example traces of evoked PSs during input/output (I/O) function and paired-pulse stimulation (PPR) in DMS and DLS. Extended Data Figure 4-2 shows that field potentials were robustly blocked by CNQX (10 μm), indicating that these recordings primarily reflect activation of AMPA receptors. Calibration: 0.2 mV, 2 ms. Values are presented as mean (±SEM), **p < 0.01, *p < 0.05 significant as compared with nicotine and saline rats (*n* = x/y; x: number of recordings/y: number of rats recorded).

Field potential recordings were performed as previously described (Adermark et al., 2011). Briefly, local field population spikes (PSs) were activated with a frequency of 0.05 Hz in subregions of the: ventral striatum nucleus accumbens core (NAcC) and shell (NAcSh); dorsal striatum [dorsomedial striatum (DMS); dorsolateral striatum (DLS)]; amygdala [central nucleus of the amygdala (CeA) and basolateral amygdala; Beridze et al., 2020; Figs. 2*A*,*F*, 3*A*, 4*A*,*F*; Extended Data Figs. 2-1, 3-1, 4-1]. Only rats that obtained ≥15 infusions in the last 10 self-administration sessions were considered in the electrophysiological recordings. Recordings in different brain areas were conducted in separate brain slices retrieved from the same rat, thereby minimizing the risk of individual variation between brain regions and treatments. Stimulation electrodes (World Precision Instruments; type TM33B) were positioned locally, 0.2–0.3 mm from the recording electrode (borosilicate glass, 2.5–4.5 MΩ, World Precision Instruments; Extended Data Figs. 2-1, 3-1, 4-1), and the amplitude of the population spike (PS) were measured. Signals were amplified with a custom-made amplifier, filtered at 3 kHz, and digitized at 8 kHz.

10.1523/ENEURO.0468-22.2023.f2-1 Extended Data Figure 2-1 ***A***, Position of recording and stimulation electrodes in BLA and (***D***) CEA. ***B***, Example traces show evoked PSs during input/output (I/O) function in BLA and (***E***) CEA. ***C***, Response amplitude during paired-pulse stimulation (PPR) in BLA and (***F***) CEA. Calibration: 0.2 mV, 2 ms. Download Figure 2-1, TIF file.

10.1523/ENEURO.0468-22.2023.f3-1 Extended Data Figure 3-1 ***A***, Position of recording and stimulation electrodes in NAcC and (***D***) NAcSh. ***B***, Example traces show evoked PSs during input/output (I/O) function in NAcC and (***E***) NAcSh. ***C***, Response amplitude during paired-pulse stimulation (PPR) in NAcC and (***F***) NAcSh. Calibration: 0.2 mV, 2 ms. Download Figure 3-1, TIF file.

10.1523/ENEURO.0468-22.2023.f4-1 Extended Data Figure 4-1 ***A***, Position of recording and stimulation electrodes in DMS and (***D***) DLS. ***B***, Example traces show evoked PSs during input/output (I/O) function in DMS and (***E***) DLS. ***C***, Response amplitude during paired-pulse stimulation (PPR) in DMS and (***F***) DLS. Calibration: 0.2 mV, 2 ms. Download Figure 4-1, TIF file.

10.1523/ENEURO.0468-22.2023.f4-2 Extended Data Figure 4-2 ***A***, Electrophysiological field potential recordings demonstrated a robust depression elicited by the AMPA receptor antagonist CNQX (10 μm) on evoked field potentials. ***B***, Example traces show evoked PSs during baseline (blue) and after CNQX perfusion after 5, 10, 20, 30 min in the DLS. Calibration: 0.2 mV, 2 ms. Download Figure 4-2, TIF file.

During field potential recordings, slices were perfused with prewarmed aCSF (30°C) containing the GABAA receptor antagonist bicuculline to isolate excitatory transmission to monitor putative effects by treatment on neurotransmission, stimulus/response curves were created by stepwise increasing the stimulation strength, and by monitoring the paired pulse ratio (PPR). For PPR, responses were evoked with a paired pulse stimulation (50-ms interpulse interval) at approximately half max stimulation strength, and PPR was calculated by dividing the second pulse (PS2) with the first pulse (PS1).

### Blinding and randomization

The laboratory animals were randomly assigned to the different experimental groups considering their nicotine and saline responding baseline. Data collection and analysis of electrophysiological measures were performed blinded between the groups.

### Data and statistical analysis

Data are expressed as mean ± SEM and analyzed with STATISTICA (Stat Soft 13.0 RRID: SCR_014213). Statistically significant difference was set at *p* < 0.05. Group size of *n* ≥ 5 was employed for statistical evaluation and using randomization the experimental groups were designed accordingly. Homogeneity of variance was assessed using the Levene’s test. Data did not violate the assumption of homogeneity and were therefore analyzed using parametric ANOVA, with factors for the respective analysis indicated in conjunction with its results. When appropriate, *post hoc* comparisons were performed using Newman–Keuls or Dunnetts’ test. A Pearson’s correlation analysis was used to assess the relationship between behavioral measures.

Electrophysiological data were extracted and analyzed using Clampfit version 10.2 (Molecular Devices), Microsoft Excel (Microsoft Corp), and GraphPad Prism version 9.0.1 (GraphPad Software). Gaussian distribution was tested with D’Agostino-Pearson omnibus normality test. A two-way ANOVA was used for input/output function, while paired or unpaired Student’s *t* tests were used for statistical analysis of the PPR.

A factor analysis was performed using principal component extraction followed by normalized varimax rotation of “input/output function” data. Correlation analysis between active lever presses during the cue-induced reinstatement test and the network of interest were performed using the Pearson correlation coefficient value (see Extended Data 1 for additional details in data analysis and outline of experimental approach).

10.1523/ENEURO.0468-22.2023.ed1 Extended Data 1 Extended data methods, outline of experimental approach, statistical analysis, and extended data figures. Download Extended Data 1, DOCX file.

## Results

### Nicotine self-administration and relapse to nicotine seeking

Rats in the nicotine (*n* = 19) and saline (*n* = 14) groups underwent self-administration training for 20 sessions initially in fixed ratio-1 (FR1; 7 d) and then fixed ratio-3 (FR-3; 13 d) schedule of reinforcements (Fig. 1*A*). We decided to use saline self-administering rats and not nicotine yoked to control the contribution of operant responding elicited by cue-contingency. Rats within the nicotine group achieved a similar number of nicotine infusions (≤20% variation in total responses over the last seven sessions) indicating that the amounts of nicotine on-board were similar before entering the abstinence phase. Overall ANOVA revealed a significant effect of group (*F*_(1,31)_ = 69.80, *p* < 0.0001), session (*F*_(19,589)_ = 3.85, *p* < 0.0001) and group × session interaction (*F*_(19,589)_ = 10.99, *p* < 0.0001) highlighting a significantly higher number of self-infusions in the nicotine rats compared with the saline group. *Post hoc* Newman–Keuls analysis showed a significant difference on days 8–20 (*p* < 0.001) between the two groups (Fig. 1*B*). Analysis of lever responses over training demonstrated a significant effect of group (*F*_(1,31)_ = 62.64, *p* < 0.0001), lever (*F*_(1,31)_ = 62.64, *p* < 0.0001), session (*F*_(19,589)_ = 22.83, *p* < 0.0001) and group × lever × session interaction (*F*_(19,589)_ = 20.75, *p* < 0.0001). The number of active responses over training was significantly higher in the nicotine group compared with saline animals (*p* < 0.001). Both nicotine and saline rats were able to discriminate between the levers mediating the active and inactive response showing some intrinsic reinforcing properties of the cue on its own in driving self-administration behavior (lever × group: *F*_(1,31)_ = 103.41, *p* < 0.0001; *post hoc* Newman–Keuls saline_active_ vs _inactive_, *p* = 0.0001; nicotine_active_ vs _inactive_ *p* = 0.0002; Donny et al., 2003). Inactive lever responses were very low without differences between the two groups (nicotine_inactive_ vs saline_inactive_ *p* = 0.3784; Fig. 1*C*).

Cue-induced nicotine seeking after forced abstinence was tested at two different time points (14- and 28-d abstinence). Factorial ANOVA analysis yielded significant group differences on reinstatement (*F*_(1,29)_ = 23.14, *p* < 0.0001), but found no significant effect of session (*F*_(1,29)_ = 0.86, *p* = 0.3591) and no significant “group × session” interaction (*F*_(1,29)_ = 0.02, *p* = 0.8971). Rats trained with nicotine exhibited a significant reinstatement level as compared with rats trained with saline after 14 d (*p* = 0.0070) and 28 d (*p* = 0.0044) of abstinence. Within the nicotine animals, similar levels of responding in cue-induced nicotine seeking were observed in the 14-d abstinence and 28-d abstinence group (*p* = 0.5754; Fig. 1*D*). Importantly, before reinstatement, there was no difference in baseline responding during training (average last 5 d) in the 14- and 28-d abstinence in both nicotine and saline groups (Extended Data Fig. 1-1).

We measured the responding over the course of the cue-induced reinstatement session as active lever presses over time (1-min bins of time) comparing 14- to 28-d abstinence period. Both groups reduced lever responding toward the end of the reinstatement session compared with the beginning, without entirely extinguishing the number of the active lever presses. When comparing lever responding in the first 10 min (1−10´) versus the last 10 min (111−120´) overall ANOVA revealed a significant effect of time (*F*_(1,17)_ = 27.66, *p* = 0.0001), but not abstinence group (*F*_(1,17)_ = 0.85, *p* = 0.3697) and time × abstinence group interaction (*F*_(1,17)_ = 3.31, *p* = 0.0865; Extended Data Fig. 1-2).

Interestingly, cue-induced reinstatement for nicotine positively correlated with the number of responses during nicotine training (average last 4 d of self-administration; *R*^2^ = 0.53, *p* = 0.0203; Fig. 1*E*). No significant correlation between responding during training and cue-induced reinstatement was observed in the saline group (*R*^2^ = 0.43, *p* = 0.1235).

Within the nicotine group the correlation was not dependent by the time of abstinence as the two-time points 14 and 28 d present similar interpolation curves which did not reach significance when analyzed independently (14-d abstinence group: *R*^2^ = 0.35, *p* = 0.0924; 28-d abstinence group: *R*^2^ = 0.28, *p* = 0.1116).

### Subregion-specific recruitment of amygdalo-striatal circuits in abstinence-induced nicotine seeking

The overall aim with this study was to outline neurophysiological correlates of abstinence-induced nicotine seeking behavior. To this end, electrophysiological field potential recordings were performed in subregions of the amygdala, nucleus accumbens, and dorsal striatum in brain slices from the same set of animals. Field potentials were robustly blocked by CNQX (10 μm), indicating that these recordings primarily reflect activation of AMPA receptors (Extended Data Fig. 4-2).

The two amygdaloid nuclei are tightly interconnected and sensory information from cortical areas is transferred from the BLA through glutamatergic projections to the CeA (Janak and Tye, 2015). After 14 d of abstinence, we did not find differences in the input/output function when comparing nicotine and saline rats in neither the CeA (14-d_main effect group_: *F*_(1,34)_ = 0.00, *p* = 0.9891) nor the BLA (14-d_main effect group_: *F*_(1,36)_ = 0.63, *p* = 0.4315; Fig. 2*B*,*G*). However, after protracted abstinence from nicotine we found a significantly altered input/output function in both amygdalar nuclei [CeA (28-d_main effect group_: *F*_(1,37)_ = 6.16, *p* = 0.0178); BLA (28-d_main effect group_: *F*_(1,32)_ = 7.86, *p* = 0.0085); Fig. 2*D*,*J*]. Compared with saline-exposed animals, evoked potentials in brain slices from nicotine rats were robustly depressed, and a concomitant increase in PPR was observed in the BLA (28 d: *t*_(30)_ = 2.32, *p* = 0.0333; Fig. 2*K*).

Recordings performed in NAc showed that evoked PS amplitudes in the accumbens core were significantly depressed at higher stimulation strengths after 14-d abstinence, and then more robustly at 28-d abstinence from nicotine compared with saline animals (14-d_stimulation intensity × group_: *F*_(6,252)_ = 8.05, *p* = 0.0001, *post hoc* analysis _(nicotine vs saline)_: *p* = 0.0302_(s.i. 48μA)_; 28-d_main effect group_: *F*_(1,30)_ = 9.45, *p* = 0.0045; Fig. 3*B*,*D*). PPR was not significantly affected by nicotine abstinence at any time point (14 d: *t*_(38)_ = 1.02, *p* = 0.3159; 28 d: *t*_(30)_ = 0.08, *p* = 0.9497; Fig. 3*C*,*E*). In the NAcSh stimulus response curves produced no significant differences in the amplitude of evoked field potentials in rats withdrawn from nicotine for 14 or 28 d compared with saline group (14-d_main effect group_: *F*_(1,27)_ = 1.26, *p* = 0.2673; 28-d_main effect group_: *F*_(1,35)_ = 0.22, *p* = 0.6467; Fig. 3*G*,*J*). There was also no effect influenced by treatment on PPR (14 d: *t*_(27)_ = 0.45, *p* = 0.6560; 28 d: *t*_(36)_ = 0.83, *p* = 0.4149; Fig. 3*H*,*K*).

Despite being functionally segregated, the DMS and DLS present overlapping and competing mechanisms in controlling motivated behaviors (Bergstrom et al., 2018; Vandaele et al., 2019). Abstinence from nicotine induced a significant depression of excitatory neurotransmission in the DMS that was present only at 28-d abstinence (14-d_main effect group_: *F*_(1,35)_ = 1.88, *p* = 0.1791; 28-d_main effect group_: *F*_(1,38)_ = 5.76, *p* = 0.0213; Fig. 4*B*,*D*). A significantly increased PPR in nicotine animals, reflecting a reduced release probability, was present at 14-d abstinence but not at 28-d abstinence (14 d: *t*_(38)_ = 2.29, *p* = 0.0279; 28 d: *t*_(35)_ = 0.61, *p* = 0.5444; Fig. 4*C*,*E*). In the DLS, evoked responses were significantly depressed at higher stimulation strengths after 14-d abstinence, and then more robustly at 28-d abstinence (14-d_stimulation intensity × group_: *F*_(6,180)_ = 8.04, *p* = 0.0001, *post hoc* analysis _(nicotine vs saline)_: *p* = 0.0391_(s.i. 48μA,)_; 28-d_main effect group_: *F*_(1,30)_ = 8.81, *p* = 0.0058; Fig. 3*G*,*J*). No differences were found when looking at the PPR measure (14 d: *t*_(30)_ = 1.19, *p* = 0.2441; 28 d: *t*_(25)_ = 1.25, *p* = 0.2245; Fig. 4*H*,*K*).

### Parallel rewiring of abstinence-responsive brain structures

**Figure 5.**
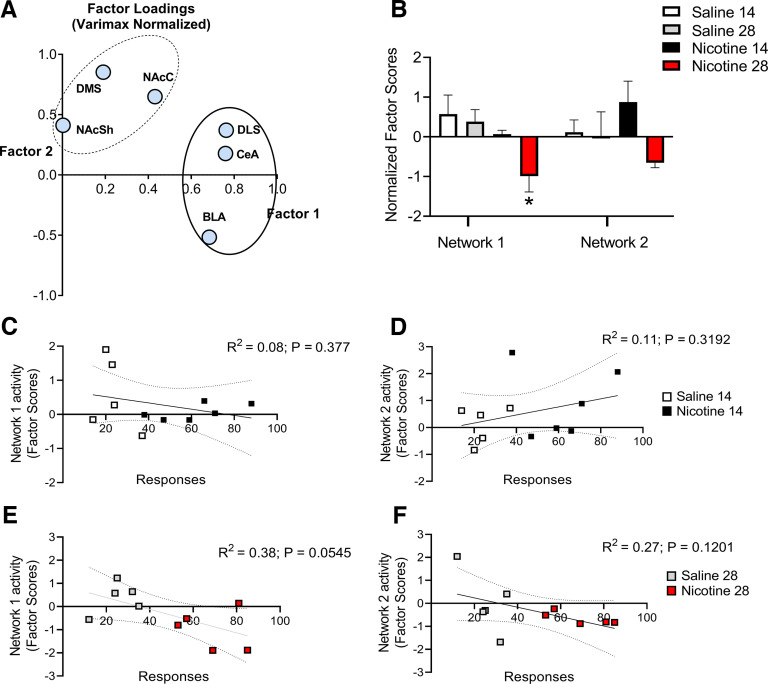
Parallel rewiring of abstinence-responsive brain structures. ***A***, A factor analysis was performed using principal component extraction followed by normalized varimax rotation on the field potential “input/output function” data. The analysis identified two networks. Responses of BLA, CeA, and DLS loaded on “network 1,” which accounted for 30% of total variance in activity, while DMS, NAcC, and NAcSh on “network 2,” which accounted for 29% of variance. ***B***, Activity of Network 1 was decreased in the nicotine 28-d abstinence group compared with rats in early abstinence and saline exposed control. ***C***, ***D***, Neither Network 1, nor Network 2 showed significant correlation with the total number of the active lever presses in cue-induced reinstatement responding in the nicotine 14-d abstinence group compared with saline rats. ***E***, ***F***, Activation of Network 1, but not Network 2, showed a close to significant correlation with the total number of the active lever presses in cue-induced reinstatement responding in the nicotine 28-d abstinence group compared with saline rats. Extended Data Table 5-1 shows the summary of correlation analysis between abstinence induced nicotine seeking and the field potential “input/output function” recorded in BLA, CeA, DMS, DLS, NAcC, and NAcSh. Values are presented as mean (±SEM), *p < 0.05 significant as compared with nicotine 28-d abstinence (*n* = 5) versus nicotine 14-d (*n* = 6) abstinence and saline 28-d (*n* = 5) and 14-d (*n* = 5) abstinence rats.

Next, based on the region-specific neuroadaptations, we wanted to further examine correlated brain structures responsive to abstinence. To this end, we conducted a factor analysis that was performed using principal component extraction followed by a normalized varimax rotation on the field potential “input/output function” data recorded for the various brain regions under analysis (BLA, CeA, DMS, DLS, and NAc core and shell). The input/output function data were plotted for both the saline and the nicotine rats to control for changes merely linked to lever pressing itself in the absence of the primary reward. Data from rats with complete recordings in all brain regions that underwent 14-d (saline *n* = 5, nicotine *n* = 6) and 28-d (saline *n* = 5, nicotine *n* = 5) abstinence were analyzed, and two factors with eigenvalues > 1 were obtained. Following normalized varimax rotation, the analysis identified two independent networks. Input/output responses of DLS, CeA, and BLA loaded on “network 1,” which accounted for 30% of the total variance, where activity of DLS and CeA accounted for >70% of the variance. DMS and NAc (core and shell) loaded on “network 2,” which accounted for 29% of the total variance with DMS accounting for >70% of the variance (Fig. 5*A*). Activity of Network 1 was differently regulated among the different groups (network 1, _main effect group_ *F*_(3,17)_ = 4.19, *p* = 0.0215). Dunnett’s *post hoc* test showed a significant decrease of activity of network 1 in the group that underwent nicotine abstinence for 28 d from nicotine compared with saline control (*p* = 0.0126; Fig. 5*B*). Activity of Network 2 did not differ significantly between groups (network 2, _main effect group_ *F*_(3,17)_ = 2.03, *p* = 0.1466; Fig. 5*B*).

10.1523/ENEURO.0468-22.2023.tab5-1 Extended Data Table 5-1 Summary of correlation analysis between abstinence induced nicotine seeking and the field potential “input/output function” data recorded in BLA, CeA, DMS, DLS, NAcC, and NAcSh. Download Table 5-1, DOCX file.

We then performed correlation analysis between the total number of the active lever presses during the cue-induced reinstatement test in the rats that underwent nicotine abstinence for 28 d and the activity of the two networks using the Pearson correlation coefficient value. As expected, activation of Network 1, but not Network 2 showed a correlation close to significant with the total number of the active lever presses in cue-induced reinstatement responding (Network 1: *R*^2^ = 0.38, *p* = 0.0545; Network 2: *R*^2^ = 0.27, *p* = 0.1201, respectively; Fig. 5*E*,*F*), suggesting that the BLA, CeA, and DLS might contribute to cue-induced reinstatement at later abstinence points. There were not significant correlations between activity of network 1 or 2 and 14-d nicotine seeking (*R*^2^ = 0.23, *p* = 0.1553 and *R*^2^ = 0.28, *p* = 0.4173, respectively; Fig. 5*C*,*D*).

## Discussion

The present study outlines progressive neuroadaptations within amygdalo-striatal neurocircuitries in abstinence-induced nicotine seeking. Electrophysiological recordings from six different brain regions in the same set of male rats showed temporal and region-specific suppression of evoked glutamatergic transmission after 14- and 28-d abstinence in subjects with a history of extended nicotine self-administration. While the data presented here show that many brain regions are recruited during nicotine abstinence, a network entailing the DLS, CeA, and BLA appeared to be especially associated with reinstatement responding suggesting a possible contribution of the observed neuroplasticity in nicotine seeking behavior at later abstinence points.

### Cue-induced reinstatement of nicotine seeking after 14 and 28 d of abstinence

In humans, relapse to smoking is most common within the first few weeks after smoking cessation and a similar time-related reinstatement of nicotine seeking behavior has been also reported in rodents (Hughes et al., 2004; Funk et al., 2016; Markou et al., 2018). However, cue-induced craving may increase with abstinence duration, as cigarette smokers show a higher level of craving after 35-d abstinence than that after one week (Bedi et al., 2011). The data presented here show that environmental conditioning cues evoke the same magnitude of reinstatement to nicotine at both 14 and 28 d of abstinence without observing an incubation effect. This is in line to the inverted U-shaped curve of incubation of nicotine seeking showing that the scale of responding during reinstatement follows a similar course when considering 14 and 28 d of abstinence (Funk et al., 2016). Moreover, we found that reinstatement levels correlated positively with the response rates during nicotine self-administration. These results confirm previous observations indicating that consummatory behavior of nicotine, as also of other psychostimulants at doses known to induce pharmacological effects, is critical for reinstatement to drug seeking (Ball et al., 2007; X. Liu et al., 2008).

### Abstinence-induced amygdalo-striatal neuroadaptations

One month of nicotine self-administration followed by abstinence-induced nicotine seeking produced a suppression of evoked glutamatergic transmission in dorsal and ventral striatum at an early stage, while a hypoglutamatergic state in subregions of the amygdala occurred only after 28-d abstinence. The observed neuroadaptations were most likely a product of the synergistic interaction between nicotine and salient cues and not a distinctive contribution of Pavlovian learning per se as we used a control group that self-administered saline at the same experimental conditions as the nicotine group.

Both BLA and CeA have been shown to exert control over habitual seeking behavior in rats (Murray et al., 2015) and to be part of a brain network that encodes for incubation of drug craving (Pickens et al., 2011). Following 28-d abstinence, evoked field potentials were significantly decreased in both the CeA and BLA with the latter accompanied by an increase in PPR. While many factors may underly an increased response to a second test stimulus, the increase in PPR suggests that neuroadaptations in BLA, but not CeA, are connected to a reduced probability of transmitter release (Morales et al., 2018; Vázquez-Vázquez et al., 2022; Vestin et al., 2022). Synaptic depression of BLA may further affect neurotransmission in both CeA and NAc and therefore alter the valence encoding of the nicotine-associated cues (Beyeler et al., 2018). A more pronounced change in glutamate adaptation seen at a later abstinence period within the amygdala might reflect a more pronounced negative emotional valence although the behavioral outcome was the same after 14- and 28-d abstinence. This is supported also by previous findings with psychostimulants, showing the role of the CeA in cue-induced reinstatement at more prolonged forced abstinence (Lu et al., 2005; Thiel et al., 2012; Li et al., 2015).

It should be noted that the data recorded here reflect sustained changes in neurotransmission during abstinence conditions, which may not correlate with the activity of neuronal ensembles during cue-induced reinstatement testing. Furthermore, we cannot fully exclude that the single reinstatement session might have contributed to the changes observed. However, at the two different abstinence points, time-selective and brain region-selective changes in glutamatergic transmission were observed, despite similar reinstatement levels. This suggests that the observed neuroadaptations rely mainly on the abstinence period rather than the reinstatement itself.

In striatal subregions, neuroadaptations were observed in the DMS, DLS, and NAcC at 14-d abstinence, and persisted after one month. Neurotransmission in the NAc shell was not affected by abstinence at any of the time-points considered. The observed subregion-specific effects could be because of a distinct recruitment of the two accumbal subregions in response to Pavlovian cues with NAc core playing a role in incubation of drug craving (Ito et al., 2004; Martin et al., 2006; Nall et al., 2021). Within the dorsal striatum, we observed a progressive depression of synaptic activity in both DMS and DLS. Decreased excitatory neurotransmission in the striatum is most likely of clinical relevance as the inability to disengage from habitual associations when the contingency is disrupted has been shown to be related to a significant reduction in glutamate concentration in the putamen in cocaine addicted patients (Ersche et al., 2021). While recordings performed in striatal subregions primarily supported nicotine-induced transformations in postsynaptic neurons, PPR was significantly increased in the DMS after 14-d abstinence, indicative of a reduced probability of transmitter release (Meneses et al., 2015; Vázquez-Vázquez et al., 2022). It is relevant to note the PPR is measured at around half max of the response amplitude. At this lower stimulation intensity, a decrease in PS amplitude was also noted in the DMS, indicating that synaptic output may be depressed especially at lower synaptic activity.

### A hypoglutamatergic state during abstinence-induced nicotine seeking

The electrophysiological field potential recordings presented here primarily reflect AMPA receptor activation and demonstrated a ubiquitous depression of glutamate neurotransmission in animals with a previous history of chronic nicotine intake compared with saline-exposed rats. These findings are partially supported by previous studies showing that nicotine decreases the probability of transmitter release at glutamatergic terminals (Licheri et al., 2018) and that protracted abstinence leads to synaptic depression in striatal subregions (Adermark et al., 2016; Morud et al., 2016). The neuroadaptations described here were primarily linked to postsynaptic transformations and may be associated with alterations in the expression of glutamate receptors and/or transporters (Kenny et al., 2009; Knackstedt et al., 2009; D'Souza and Markou, 2013; Alasmari et al., 2021).

Importantly, while reinstated nicotine-associated cues are known to produce a transient increase in extracellular glutamate release in the accumbens limited to the relapse (Gipson et al., 2013), it is not clear whether alterations of glutamatergic signaling during abstinence sustain nicotine seeking behaviors. In light of this, we performed our neurophysiological assessments 2 d after the reinstatement session to avoid putative effects on glutamatergic transmission produced by the session per se Glutamate synaptic activity might as well be affected by extinction training before reinstatement that elicits neurotransformations reflecting learning processes related to the extinction experience (Bouton et al., 2006; Knackstedt et al., 2010). However, from a clinical perspective, extinction during abstinence is not a condition experienced by human smokers before relapse. Therefore, our findings implying forced abstinence could represent a more accurate representation of the human condition.

### A brain network associated with cue-induced reinstatement of nicotine seeking

Principal component analysis showed that electrophysiological field potential recordings segregated in two different brain networks. Specifically, the neuroadaptations that occurred in the DMS clustered together with the changes in the subregions of the nucleus accumbens. This is partially in line with the neuroanatomical evidence where the DMS and nucleus accumbens share both glutamatergic projections from cortical subregions as well as dopaminergic inputs from the ventral tegmental area (Barker et al., 2016). However, the glutamatergic activity of this network did not differ significantly between the groups and did not show any correlation with cue-induced nicotine seeking behavior.

Interestingly, DLS formed a network with the subregions of the amygdala. The activity of this network differed between treatment groups and abstinence time-points. Network activity correlated close to significance (*p* = 0.0545) with relapse-like behavior after 28 d of nicotine abstinence which was associated with total nicotine exposure. This suggest that activity of the DLS-amygdala network is related to nicotine seeking which in turn is affected by nicotine exposure (X. Liu et al., 2008; Butler et al., 2021).

Remarkably, PCA analysis demonstrated a strong association between neuroadaptations in CeA and DLS. This is especially interesting considering the implication of the CeA and its connection with a circuit involving DLS in the expression of stimulus-response habits (Lingawi and Balleine, 2012). Anatomically the CeA consists of GABAergic neurons resembling striatal medium spiny neurons (Sun and Cassell, 1993; J. Liu et al., 2021) but does not show direct connections with the DLS. However, amygdala might influence DLS activity through multisynaptic connections possibly involving the ventral striatum and through substantia nigra pars compacta dopaminergic neurons (Han et al., 1997; Lingawi and Balleine, 2012; Murray et al., 2015). Finally, the PCA analysis suggest that abstinence-induced nicotine seeking is driven by an interplay of neuroadaptations occurring inf multiple brain regions rather than the single region, as activity of the latter did not correlate significantly with nicotine reinstatement (Extended Data Table 5-1).

In conclusion, altogether, our findings suggest a persistent hypoglutamatergic state over protracted nicotine abstinence. The electrophysiological changes observed here reflected nicotine-cue association and not the operant learning per se, as confirmed by the absence of the effect in the saline self-administering rats. The neuroadaptation changes observed during nicotine abstinence were detected within a time window that allows therapeutic intervention rather than reflecting merely transitory mechanisms difficult to detect and promptly treat.

One limitation of the study is that only male rats were used, and the nicotine abstinence-induced neuroadaptations may differ in female rats. However, no differences have been observed during operant lever responding, extinction, or reinstatement of nicotine seeking (Feltenstein et al., 2012; Leyrer-Jackson et al., 2021). Furthermore, NMDA receptors have been shown to be important for the regulation of the reinforcing effects of nicotine in female rats, suggesting that these behaviors might be driven by the same mechanism also in females (D'Souza and Markou, 2013). Another limitation of our study is the lack of causality between the electrophysiological findings and the behavior. Future investigations will be essential to prove a functional role of the amygdala-DLS hypoglutamatergic state and nicotine seeking. Still, the identification of a brain network involving the amygdala and the DLS associated to cue-induced reinstatement of nicotine seeking has the potential to provide a basis for development of translational biomarkers (Lerman et al., 2014) in which the magnitude of a hypoglutamatergic depressive state might predict relapse to nicotine seeking, advancing clinical development of preventive strategies in nicotine addiction.

## Synthesis

Reviewing Editor: Jonathan Lee, University of Birmingham Decisions are customarily a result of the Reviewing Editor and the peer reviewers coming together and discussing their recommendations until a consensus is reached. When revisions are invited, a fact-based synthesis statement explaining their decision and outlining what is needed to prepare a revision will be listed below. The following reviewer(s) agreed to reveal their identity: NONE. Note: If this manuscript was transferred from JNeurosci and a decision was made to accept the manuscript without peer review, a brief statement to this effect will instead be what is listed below.

The menuscript was considered by one reviewer (who had previously reviewed the submission and J Neurosci) and myself as the reviewing editor. We agreed that the revisions and responses to the previous reviews have greatly strengthened the manuscript. A couple of outstanding comments remain, which we would encourage the authors to consider addressing.

It is indeed a strength of the paper that electrophysiological recordings from 6 brain regions have been conducted from the same animals. In their response to the concerns related to the PCA analysis and subsequent analysis of “network 1” and “network 2” the authors state their belief “that the observed reinstatement is driven by an interplay in activity of multiple brain regions rather than a single region”. This is, in fact, a testable hypothesis if the correlations for each brain region were compared to that of the two networks to determine if the network compilation does indeed more accurately represent the behavioral data than any one brain region.

We also suggest the data from the individual brain regions analyzed as in figure 5B-F be included in the manuscript, at least as supplementary data and the reader can draw an accurate conclusion about the usefulness of the networks proposed/identified here. Either way, the findings would be interesting to the field.
