# Supplementary figures and images for: Abstinence-Induced Nicotine Seeking Relays on a Persistent Hypoglutamatergic State within the Amygdalo-Striatal Neurocircuitry
Source: eNeuro. 2023 Feb 20;10(2):ENEURO.0468-22.2023. doi: 10.1523/ENEURO.0468-22.2023 (PMC9946069; doi:10.1523/ENEURO.0468-22.2023)

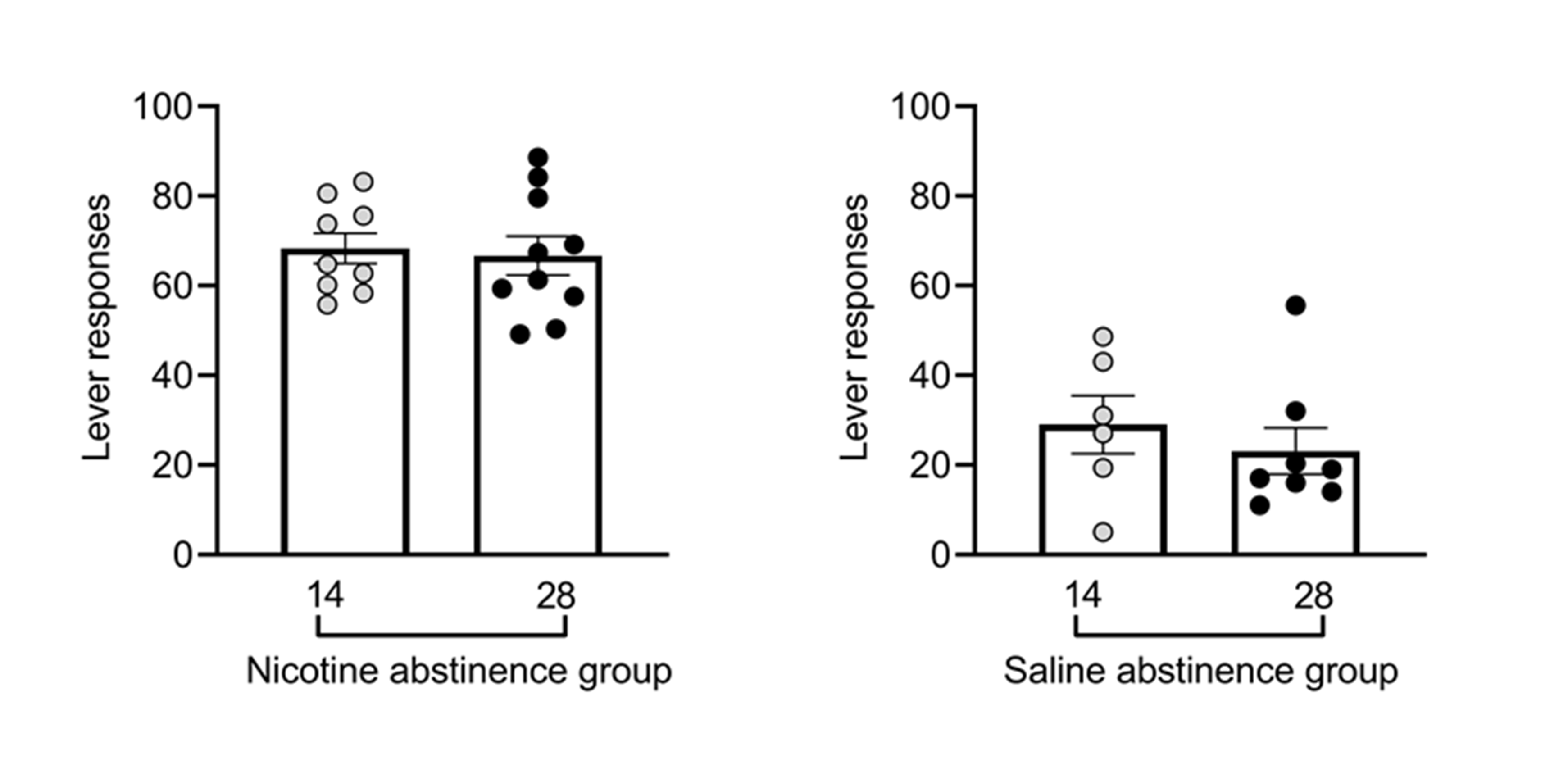

Supplement: Extended Data Figure 1-1 — Baseline responding during training (average last 5 d) for nicotine and saline rats in the 14-d abstinence and 28-d abstinence group. Student’s t test showed no significant difference in the number of active lever presses in the14-d abstinence and 28-d abstinence nicotine group (t(17) = 0.30, p = 0.77) and saline group (t(12) = 0.79, p = 0.44). Download Figure 1-1, TIF file. [file enu-eN-NWR-0468-22-s03.tif]

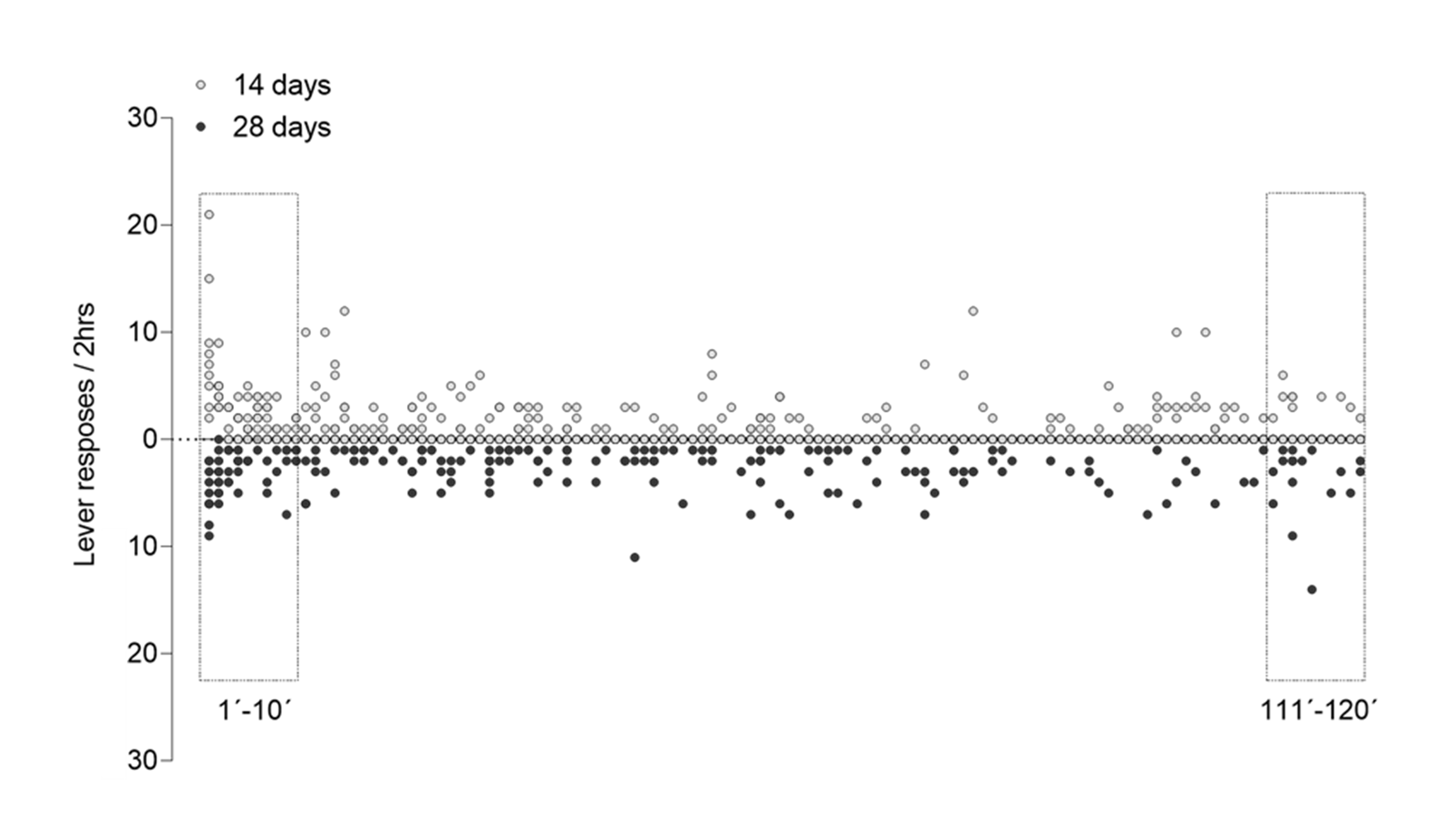

Supplement: Extended Data Figure 1-2 — Time course of active lever responding of cue-induced reinstatement of nicotine seeking (1-min bins of time) in the 14- and 28-d abstinence group. When comparing lever responding in the first 10 min (1–10´) versus the last 10 min (111–120´) overall ANOVA revealed a significant effect of time (F(1,17) = 27.66, p < 0.001), but not abstinence group (F(1,17) = 0.85, p = 0.36) and time × abstinence group interaction (F(1,17) = 3.31, p = 0.087). Download Figure 1-2, TIF file. [file enu-eN-NWR-0468-22-s04.tif]

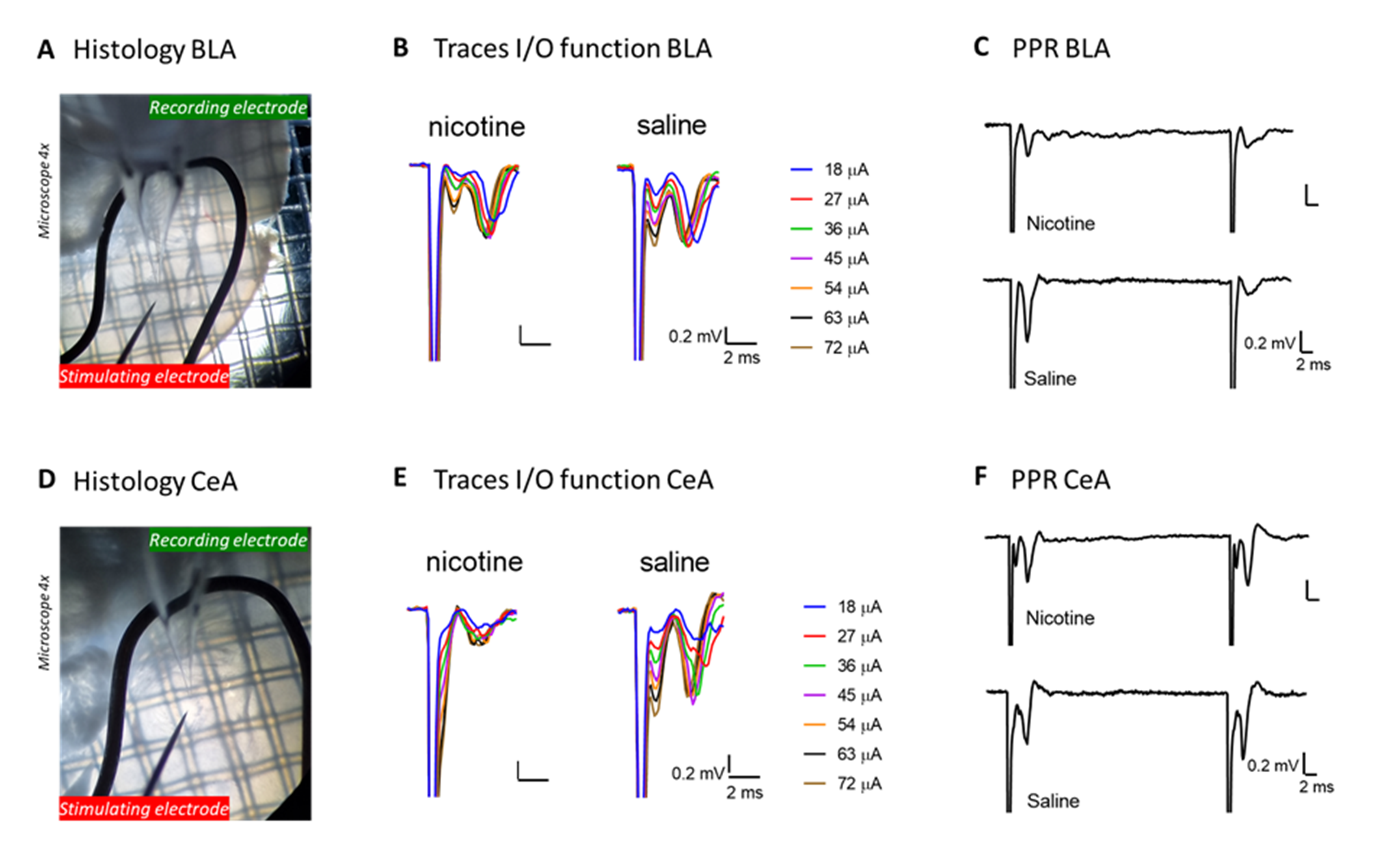

Supplement: Extended Data Figure 2-1 — A, Position of recording and stimulation electrodes in BLA and (D) CEA. B, Example traces show evoked PSs during input/output (I/O) function in BLA and (E) CEA. C, Response amplitude during paired-pulse stimulation (PPR) in BLA and (F) CEA. Calibration: 0.2 mV, 2 ms. Download Figure 2-1, TIF file. [file enu-eN-NWR-0468-22-s05.tif]

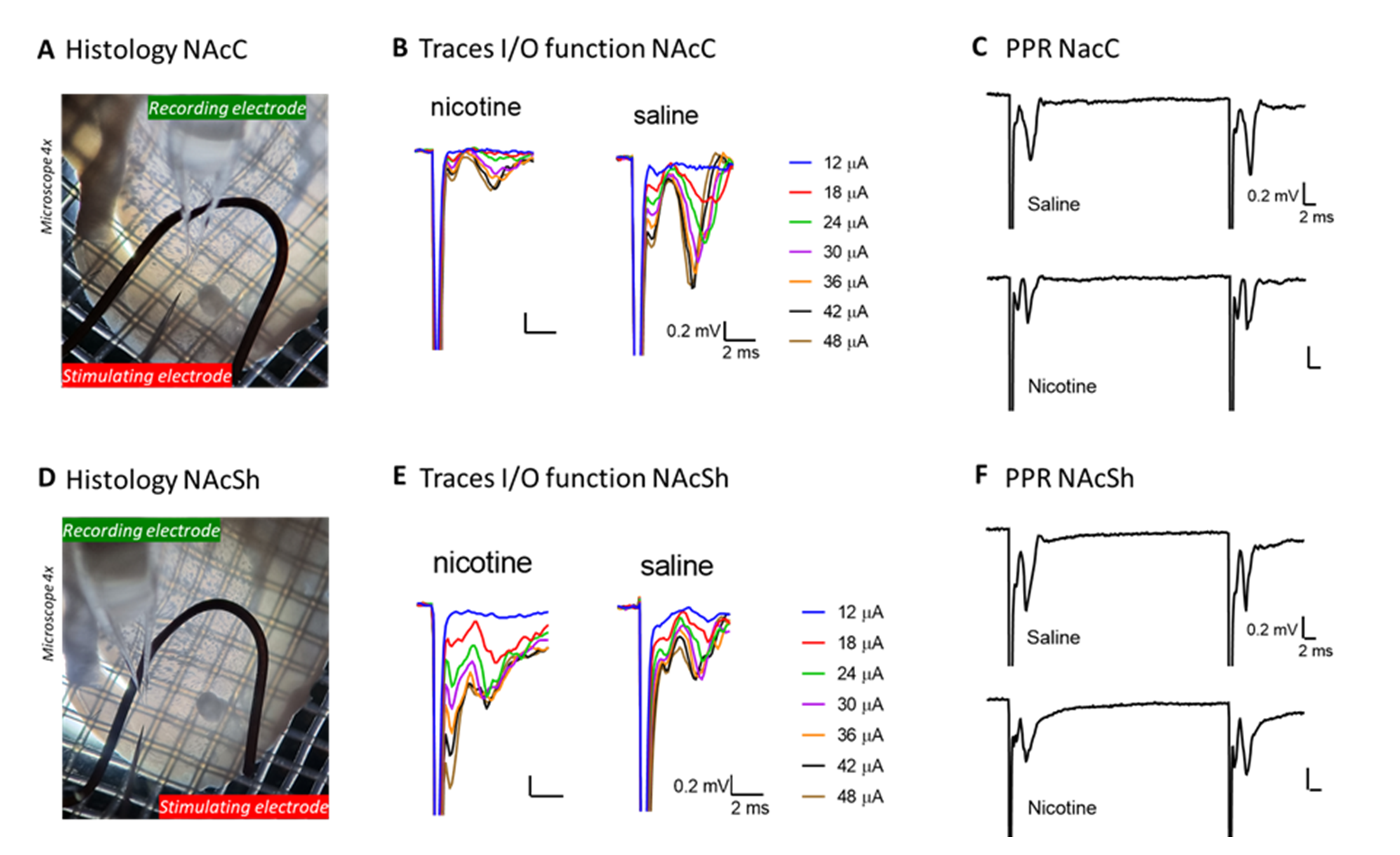

Supplement: Extended Data Figure 3-1 — A, Position of recording and stimulation electrodes in NAcC and (D) NAcSh. B, Example traces show evoked PSs during input/output (I/O) function in NAcC and (E) NAcSh. C, Response amplitude during paired-pulse stimulation (PPR) in NAcC and (F) NAcSh. Calibration: 0.2 mV, 2 ms. Download Figure 3-1, TIF file. [file enu-eN-NWR-0468-22-s06.tif]

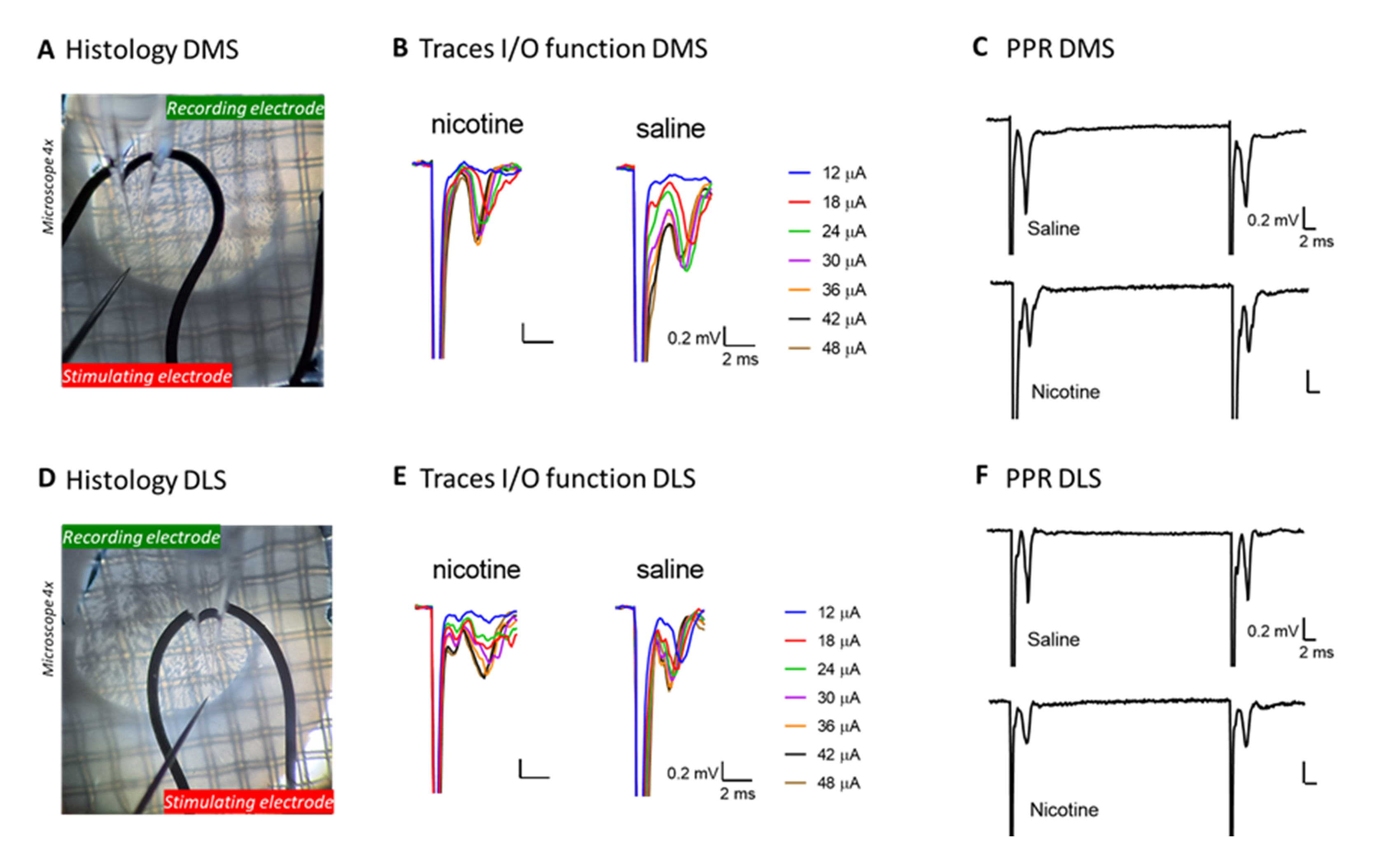

Supplement: Extended Data Figure 4-1 — A, Position of recording and stimulation electrodes in DMS and (D) DLS. B, Example traces show evoked PSs during input/output (I/O) function in DMS and (E) DLS. C, Response amplitude during paired-pulse stimulation (PPR) in DMS and (F) DLS. Calibration: 0.2 mV, 2 ms. Download Figure 4-1, TIF file. [file enu-eN-NWR-0468-22-s07.tif]

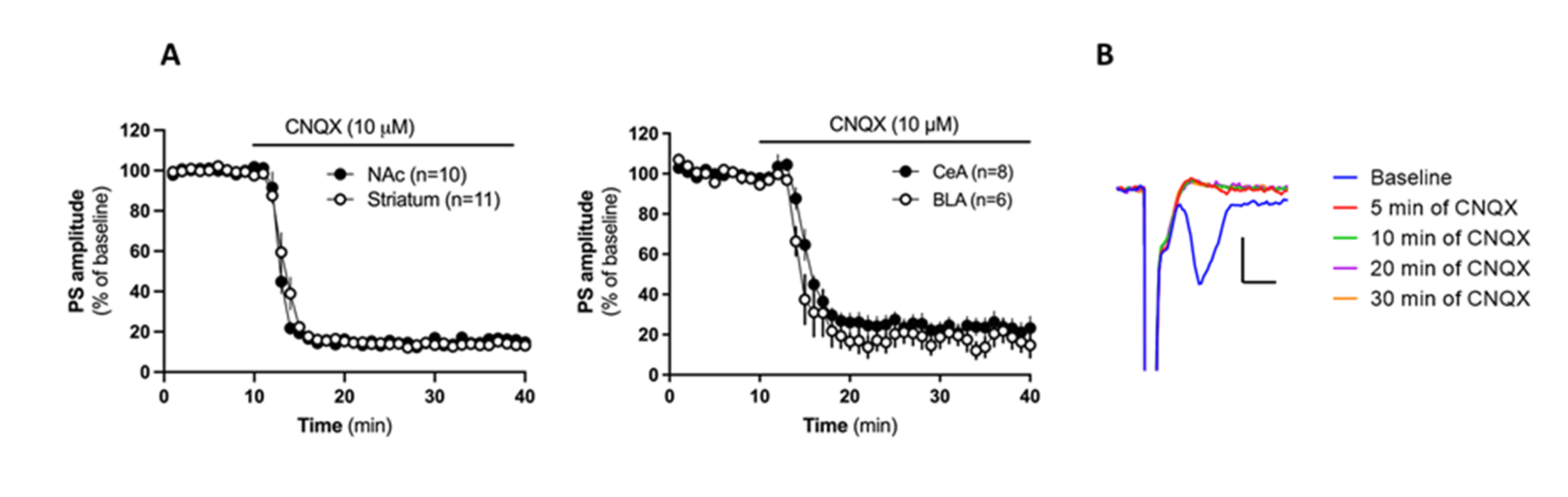

Supplement: Extended Data Figure 4-2 — A, Electrophysiological field potential recordings demonstrated a robust depression elicited by the AMPA receptor antagonist CNQX (10 μm) on evoked field potentials. B, Example traces show evoked PSs during baseline (blue) and after CNQX perfusion after 5, 10, 20, 30 min in the DLS. Calibration: 0.2 mV, 2 ms. Download Figure 4-2, TIF file. [file enu-eN-NWR-0468-22-s08.tif]
