# Supplementary material for: Abstinence-Induced Nicotine Seeking Relays on a Persistent Hypoglutamatergic State within the Amygdalo-Striatal Neurocircuitry
Source: eNeuro. 2023 Feb 20;10(2):ENEURO.0468-22.2023. doi: 10.1523/ENEURO.0468-22.2023 (PMC9946069; doi:10.1523/ENEURO.0468-22.2023)
Supplement: Extended Data 1 — Extended data methods, outline of experimental approach, statistical analysis, and extended data figures. Download Extended Data 1, DOCX file. [file enu-eN-NWR-0468-22-s02.docx]

**Abstinence-induced nicotine seeking relays on a persistent hypoglutamatergic state within the amygdalo-striatal neurocircuitry**

Ana Domi, Esi Domi, Oona Lagstrom, Francesco Gobbo, Elisabet Jerlhag, Louise Adermark.

**EXTENDED DATA 1**

**Extended Data Methods**

*Outline of experimental approach*

The experimental procedures include behavioural and neurophysiological assessments. A total of 36 rats were initially used. The training phase consisted in two different groups; saline (n = 16) and nicotine (n = 20) rats that underwent 20 days of operant behaviour self-administration at same conditions. Then rats underwent the abstinence phase and were randomly divided in four groups: nicotine 14-days abstinence (n = 9), nicotine 28-days abstinence (n = 10), saline 14-days abstinence (n = 6) and saline 28-days abstinence (n = 8). Cue-induced reinstatement of nicotine seeking was assessed in a single session for each abstinence time point. Following 48 hrs after the reinstatement session we carried out the electrophysiological recordings in each of the four groups. Recordings in different brain areas were conducted in separate brain slices from the same rats thereby minimizing the risk of individual variation between brain regions. Two rats were excluded from the study for catheter failure and one rat was removed for non-success to acquire nicotine self-administration.

*Statistical analysis*

Data are expressed as mean ± standard error with statistically significant difference set at P< .05. Group size of n ≥ 5 was employed for statistical evaluation and using randomization the experimental groups were designed accordingly. Data were examined for significant violations for assumptions of homogeneity of variance by using Levene’s test. Data did not violate the assumption of homogeneity and were therefore analyzed using parametric analysis of variance (ANOVA), followed by post hoc tests when appropriate. The number of infusions during the acquisition and maintenance of SA were analyzed by two-way ANOVA with one factor between (group) and one factor within (sessions) and the lever presses by three-way ANOVA with one factor between (group) and two factors within (lever and sessions). Cue-induced reinstatement of nicotine seeking was analyzed by two-way ANOVA with two factors between (rat group x withdrawal period). Post hoc comparisons were carried out by Newman-Keuls test or Dunnetts´ test. Statistically significant difference was set at P< 0.05. A Pearson’s correlation analysis was used to assess the relationship between nicotine intake and reinstatement of cue-induced nicotine seeking.

Electrophysiological data were analyzed using Clampfit version 10.2 (Molecular Devices, Sunnyvale, CA), Microsoft Excel (Microsoft Corp, Redmond, WA), and GraphPad Prism version 7 (GraphPad Software, San Diego, CA). Gaussian distribution was tested with D’Agostino-Pearson omnibus normality test. A 2-way ANOVA was used for comparisons over time and input/output function, while paired or unpaired Student's t tests were used for statistical analysis of the PPR.

Principal component analysis was performed using principal component extraction followed by normalized varimax rotation on the field potential “input/output function” data recorded from the various brain regions under analysis (BLA, CeA, DMS, DLS, and NAc core and shell). The input/output function data were plotted for both the saline and the nicotine rats to control for changes merely linked to lever pressing itself in the absence of the primary reward. Data from the rats that underwent 14- and 28-days abstinence were analyzed and two factors with eigenvalues > 1 were obtained. We applied normalized varimax rotation to identify the independent networks and the factor loading scores related to the “input/output function” data for each region of interest.

Correlation analysis between active lever presses during the cue-induced reinstatement test and the network of interest of the “input/output function” were performed using the Pearson correlation coefficient value.

All data were analyzed using Clampfit 10.2, Microsoft Excel, STATISTICA, Stat Soft 13.0 and GraphPad Prism 7 (GraphPad Software, San Diego, CA, USA).


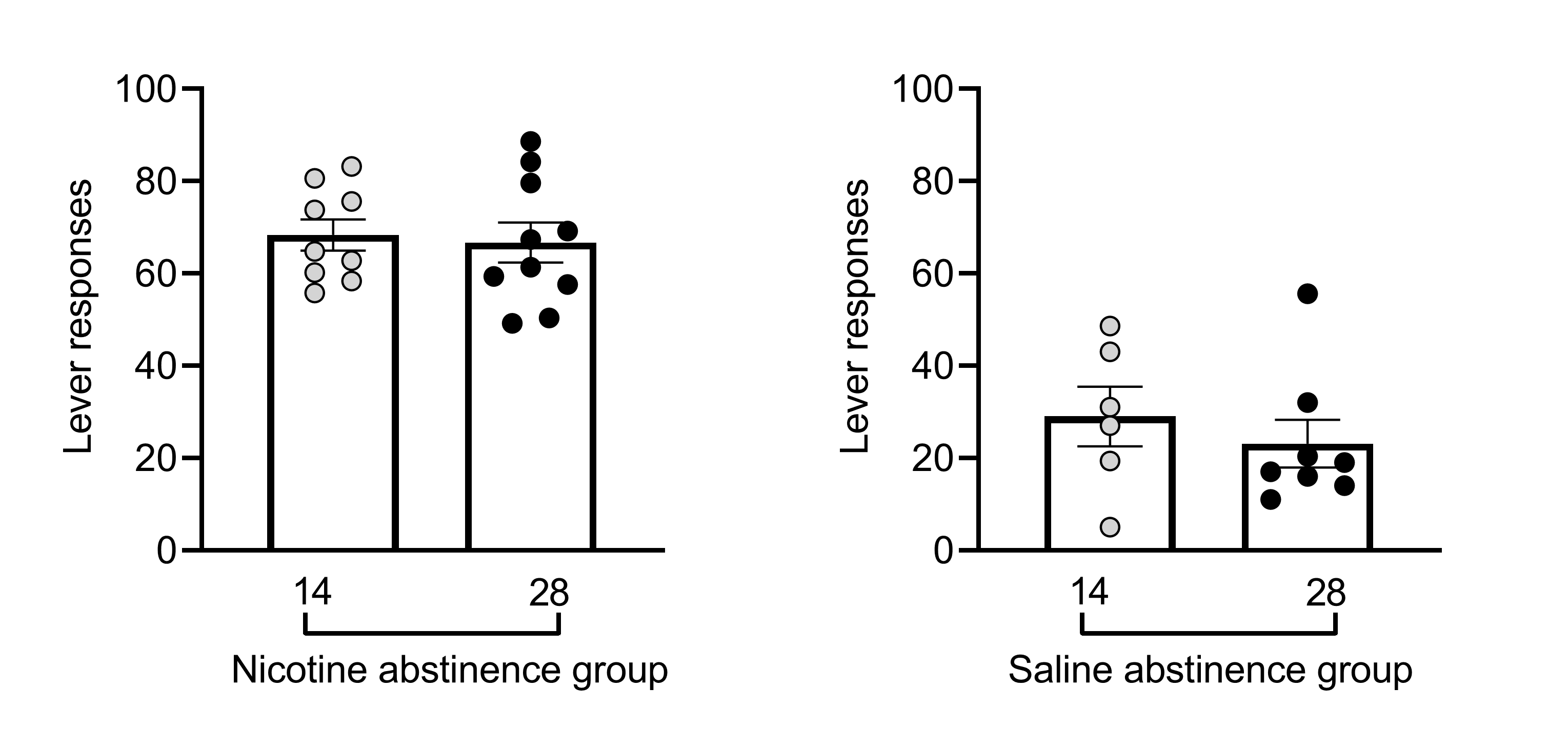
**Extended Data Figures and Legends**

**Extended Data Figure 1-1.** Baseline responding during training (average last 5 days) for nicotine and saline rats in the 14-days abstinence and 28-days abstinence group. Student’s t-test showed no significant difference in the number of active lever presses in the14-days abstinence and 28-days abstinence nicotine group (t _(17)_ = 0.30, P = 0.77) and saline group (t _(12)_ = 0.79, P = 0.44).


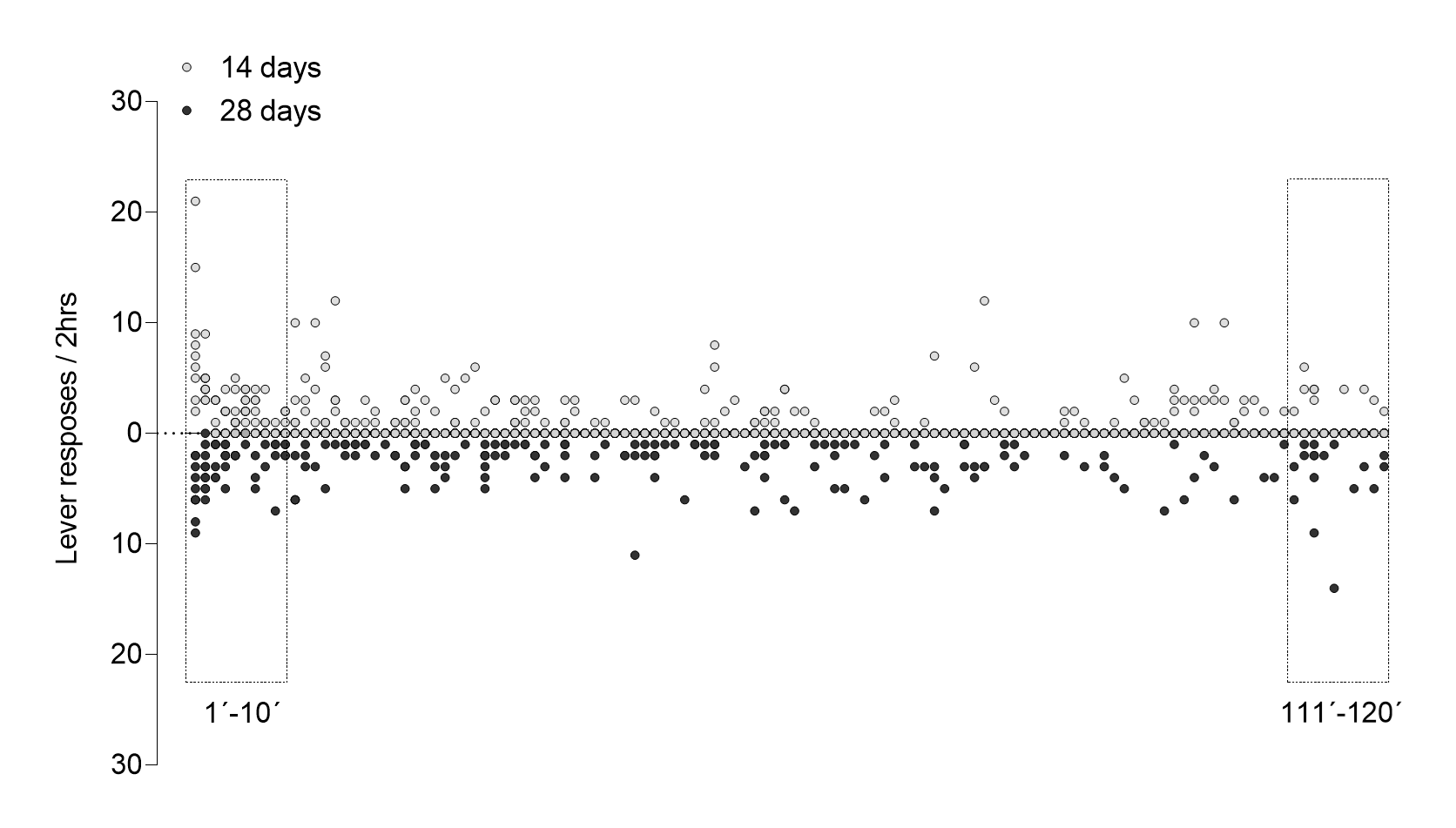


**Extended Data Figure 1-2.** Time course of active lever responding of cue-induced reinstatement of nicotine seeking (1-minute bins of time) in the 14-days and 28-days abstinence group. When comparing lever responding in the first 10 minutes (1-10´) vs the last 10 minutes (111´-120´) overall ANOVA revealed a significant effect of time (F_1,17_= 27.66, P < 0.001), but not abstinence group (F_1,17_= 0.85, P = 0.36) and time × abstinence group interaction (F_1,17_= 3.31, P = 0.087).

**
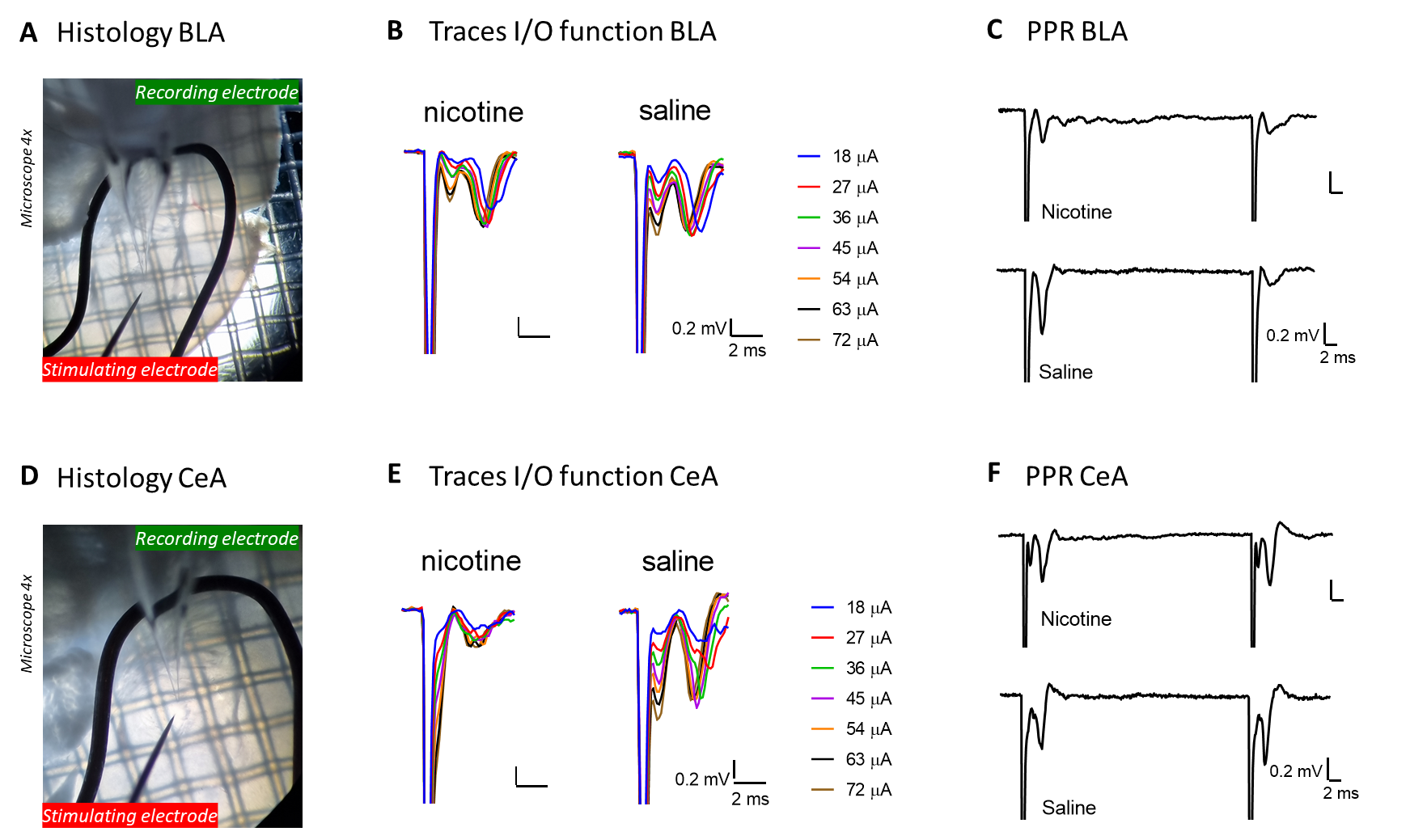
**

**Extended Data Figure 2-1.** (A) Position of recording and stimulation electrodes in BLA and (D) CEA. (B) Example traces show evoked PSs during input/output (I/O) function in BLA and (E) CEA. (C) Response amplitude during paired-pulse stimulation (PPR) in BLA and (F) CEA. Calibration: 0.2 mV, 2 ms.


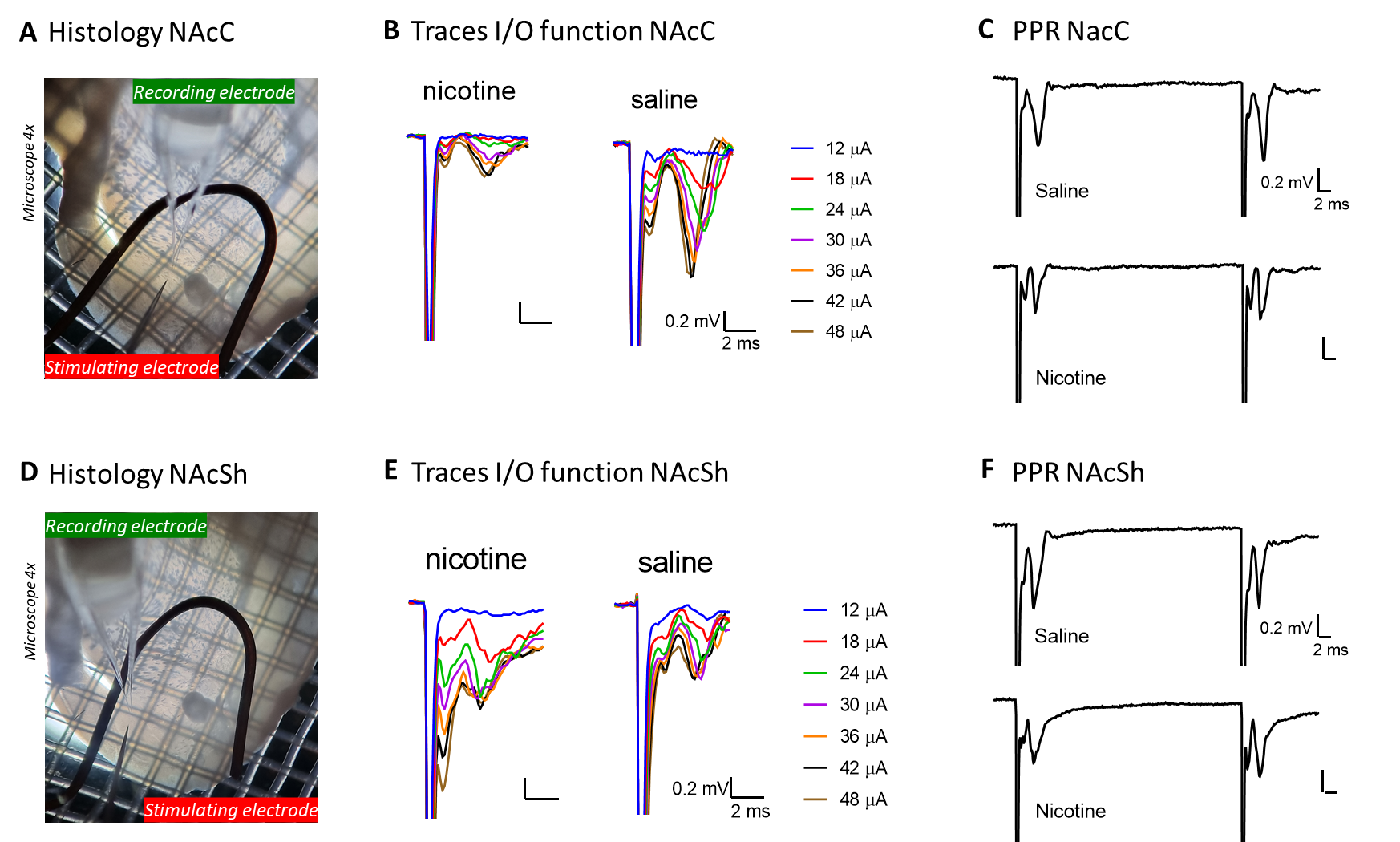


**Extended Data Figure 3-1.** (A) Position of recording and stimulation electrodes in NAcC and (D) NAcSh. (B) Example traces show evoked PSs during input/output (I/O) function in NAcC and (E) NAcSh. (C) Response amplitude during paired-pulse stimulation (PPR) in NAcC and (F) NAcSh. Calibration: 0.2 mV, 2 ms.


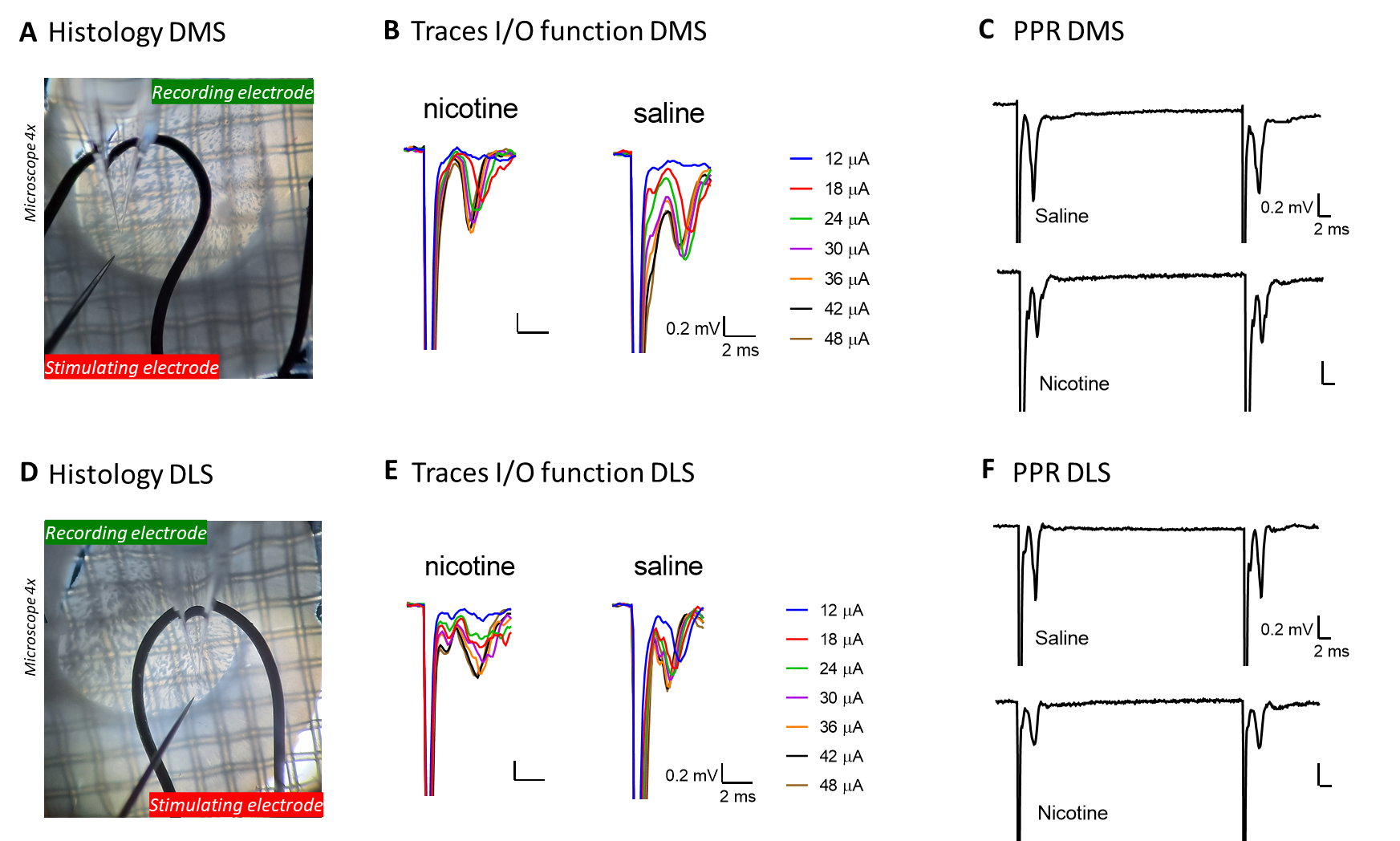


**Extended Data Figure 4-1. (**A) Position of recording and stimulation electrodes in DMS and (D) DLS. (B) Example traces show evoked PSs during input/output (I/O) function in DMS and (E) DLS. (C) Response amplitude during paired-pulse stimulation (PPR) in DMS and (F) DLS. Calibration: 0.2 mV, 2 ms.

**
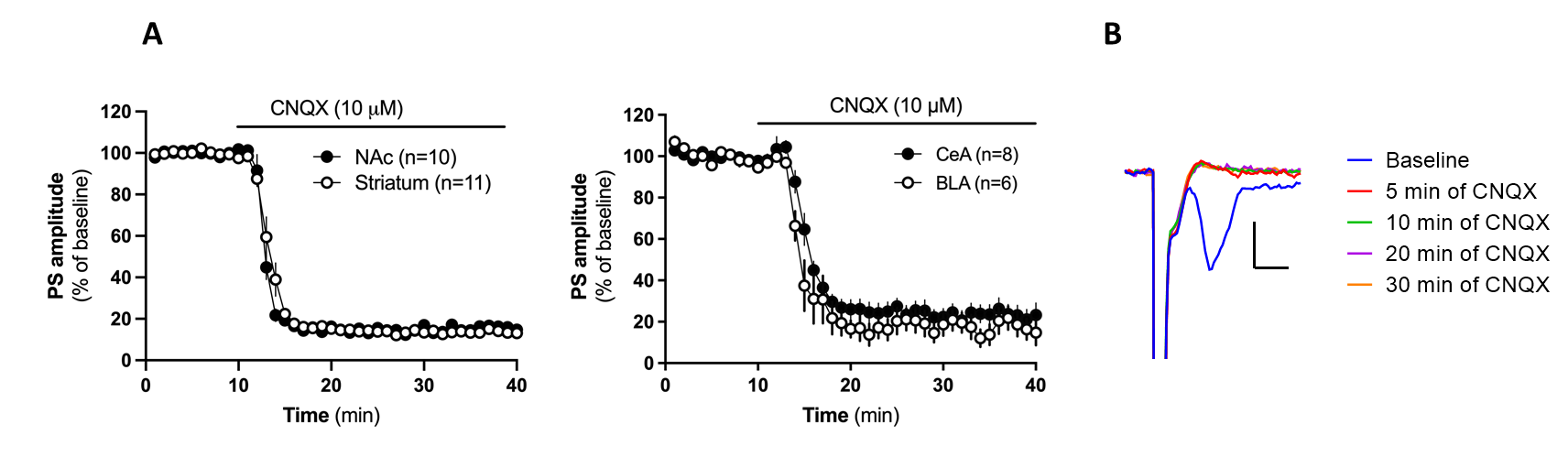
**

**Extended Data Figure 4-2.** (A) Electrophysiological field potential recordings demonstrated a robust depression elicited by the AMPA receptor antagonist CNQX (10 μM) on evoked field potentials. (B) Example traces show evoked PSs during baseline (blue) and after CNQX perfusion after 5,10,20,30 minutes in the DLS. Calibration: 0.2 mV, 2 ms.

| **Reinstatement** | **BLA** | **CeA** | **DMS** | **DLS** | **NacC** | **NacSh** |
| --- | --- | --- | --- | --- | --- | --- |
| 14-days abstinence | R = 0,0139  p = 0,7294 | R = 0,0684  p = 0,4372 | R = 0,0563  p = 0,4821 | R = 0,1327  p = 0,2708 | R = 0,0339  p = 0,5875 | R = 0,1711  p = 0,2060 |
| 28-days abstinence | R = 0,0204  p = 0,6937 | R = 0,3062  p = 0,0971 | R = 0,1244  p = 0,3175 | R = 0.3106  p = 0,0942 | R = 0,0703  p = 0,4590 | R = 0,0361  p = 0,5991 |

**Extended Data Table 5-1.** Summary of correlation analysis between abstinence induced nicotine seeking and the field potential “input/output function” data recorded in BLA, CeA, DMS, DLS, NAcC and NAcSh.
