# Supplementary material for: Abstinence-Induced Nicotine Seeking Relays on a Persistent Hypoglutamatergic State within the Amygdalo-Striatal Neurocircuitry
Source: eNeuro. 2023 Feb 20;10(2):ENEURO.0468-22.2023. doi: 10.1523/ENEURO.0468-22.2023 (PMC9946069; doi:10.1523/ENEURO.0468-22.2023)
Supplement: Extended Data Table 5-1 — Summary of correlation analysis between abstinence induced nicotine seeking and the field potential “input/output function” data recorded in BLA, CeA, DMS, DLS, NAcC, and NAcSh. Download Table 5-1, DOCX file. [file enu-eN-NWR-0468-22-s09.docx]

| **Reinstatement** | **BLA** | **CeA** | **DMS** | **DLS** | **NacC** | **NacSh** |
| --- | --- | --- | --- | --- | --- | --- |
| 14-days abstinence | R = 0,0139  p = 0,7294 | R = 0,0684  p = 0,4372 | R = 0,0563  p = 0,4821 | R = 0,1327  p = 0,2708 | R = 0,0339  p = 0,5875 | R = 0,1711  p = 0,2060 |
| 28-days abstinence | R = 0,0204  p = 0,6937 | R = 0,3062  p = 0,0971 | R = 0,1244  p = 0,3175 | R = 0.3106  p = 0,0942 | R = 0,0703  p = 0,4590 | R = 0,0361  p = 0,5991 |

**Extended Data Table 5-1.** Summary of correlation analysis between abstinence induced nicotine seeking and the field potential “input/output function” data recorded in BLA, CeA, DMS, DLS, NAcC and NAcSh.
